# Insights into the functionality of endophytic actinobacteria with a focus on their biosynthetic potential and secondary metabolites production

**DOI:** 10.1038/s41598-017-12235-4

**Published:** 2017-09-18

**Authors:** Ajit Kumar Passari, Vineet Kumar Mishra, Garima Singh, Pratibha Singh, Brijesh Kumar, Vijai Kumar Gupta, Rupak Kumar Sarma, Ratul Saikia, Anthonia O’. Donovan, Bhim Pratap Singh

**Affiliations:** 1 Molecular Microbiology and Systematics Laboratory, Department of Biotechnology, Aizawl, Mizoram University, Mizoram, 796004 India; 2 SAIF, CSIR-Central Drug Research Institute (CSIR-CDRI), Lucknow, 226012 India; 3 Department of Chemistry and Biotechnology, ERA Chair of Green Chemistry, Tallinn University of Technology, 12618 Tallinn, Estonia; 4 Biotechnology Division, CSIR-NEIST, Jorhat, Assam 785006 India; 5 Applied Biology and Biopharmaceutical Science, School of Science & Computing, Galway-Mayo Institute of Technology, Galway, Ireland

## Abstract

Endophytic actinobacteria play an important role in growth promotion and development of host plant by producing enormous quantities of novel bioactive natural products. In the present investigation, 169 endophytic actinobacteria were isolated from endospheric tissues of *Rhynchotoechum ellipticum*. Based on their antimicrobial potential, 81 strains were identified by 16rRNA gene analysis, which were taxonomically grouped into 15 genera. All identified strains were screened for their plant growth promoting attributes and, for the presence of modular polyketide synthases (PKSI, PKSII and nonribosomal peptide synthetase (NRPS) gene clusters to correlate the biosynthetic genes with their functional properties. Expression studies and antioxidant potential for four representative strains were evaluated using qRT-PCR and DPPH assay respectively. Additionally, six antibiotics (erythromycin, ketoconazole, fluconazole, chloramphenicol, rifampicin and miconazole) and nine phenolic compounds (catechin, kaempferol, chebulagic acid, chlorogenic acid, Asiatic acid, ferulic acid, arjunic acid, gallic acid and boswellic acid) were detected and quantified using UHPLC-QqQ_LIT_-MS/MS. Furthermore, three strains (BPSAC77, 121 and 101) showed the presence of the anticancerous compound paclitaxel which was reported for the first time from endophytic actinobacteria. This study provides a holistic picture, that endophytic actinobacteria are rich bacterial resource for bioactive natural products, which has a great prospective in agriculture and pharmaceutical industries.

## Introduction

The plant endosphere constitutes a complex micro-ecosystem that in several ways contributes to the development and health of the plant^1^. Endophytes are microorganisms that reside within the interior tissues of plants without exhibiting negative effects on the host plant or the environment^2^. However, some seemed to be latent pathogens and, conditionally, either induce or participate in host plant infection. Endophytes resides within medicinal plants are documented as a potential source for the production of various secondary metabolites having capacity of stress tolerance as well as to reduced disease symptoms caused by plant pathogens^3,4^. Species composition of endophytic population depends on the genotype, environmental conditions, type of soil, and growth stage of the host plant^5–14^.

Endophytic microbial communities associated within medicinal plants have been proven to have great potential as producers of novel bioactive compounds, which have a great prospective in agriculture, pharmaceutical and other industries^3,4,15^. Thus, endophytes isolation from medicinal plants has an immense significance. Endophytic actinobacteria are best known to produce a diverse range of bioactive metabolites including antimicrobial, antitumor, immunosuppressive and other pharmaceutical compounds^16^. Among endophytic actinobacteria recovered from medicinal plants, *Streptomyces* accounts for the most abundant genus^17,18^ followed by *Micromonospora*, *Actinopolyspora*, *Nocardia*, *Saccharopolyspora*, *Streptosporangium*, *Promicromonospora* and *Rhodococcus*^18,19^. Some rare genera, like *Dietzia*, *Microtetraspora*, *Actinocorallia*, *Verrucocsispora*, *Isoptericola* and *Kytococcus*^17,20^ were also reported from medicinal plants.

In search of new antibiotics against multi-drug resistant pathogens, endophytic microbes, especially actinobacteria belonging to the genus *Glycomyces* and *Streptomyces* isolated from medicinal plants, have suppressed penicillin resistant *Staphylococcus aureus*^21^. Similarly, Wang *et al*.^22^, suggested that 1-hydroxy-β-carboline, obtained from actinobacteria *Jishengella endophytica* could be a promising new hit against influenza virus type A subtype H1N1. Besides antimicrobial and antiviral potential, the endophytic actinobacteria obtained from medicinal plants also showed a significant effect as larvicidal^23^, antimalarial^24,25^, antitumor^26^, antidiabetic^27^ and also as plant growth promoters^28^.

It has been establish that the genes accountable to produce bioactive secondary metabolites were present in the genome of actinobacteria in the form of biosynthetic gene clusters^29^. Moreover, there is no reported evidence for the whole genome sequence of the actinobacteria recovered from medicinal plants. Goodfellow and Fiedler^30^, showed that the actinobacteria contain more than 20 natural product biosynthetic gene clusters responsible to produce known or expected secondary metabolites. The biosynthetic potential of actinobacteria allied with medicinal plants is estimated by the detection and expression of antimicrobial biosynthetic genes^31,32^. For expression studies, the quantitative real-time PCR (qRT-PCR) has been proved as a very highly specific and sensitive technique, as it detects very low transcript levels and can screen changes that occur during cellular differentiation^33,34^. As it is recommended, that more than one reference gene should be used to obtain accurate normalization, this work presents the expression studies of two targeted genes (PKSI and NRPS).

Hence, we investigated the multifunctional attributes of endophytic actinobacteria associated with the traditional medicinal plant *Rhynchotoechum ellipticum*, belonging to the Gesneraceae plant family from Mizoram, India. Traditionally, the leaf decoction of this plant is taken orally for the treatment of various types of cancers in Mizoram, India^35^. Our aim in the present study was to explore the endophytic actinobacteria for their biosynthetic potential by the detection and expression of NRPS and PKS genes. Our results demonstrated the existence of NRPS and PKS gene clusters within culturable endophytic actinobacteria involved in biosynthesis of secondary metabolites and noted their existence as potential source for future natural product research. The obtained strains showing multifunctional activities were genotypically characterized and the major bioactive compounds were determined and quantified using UHPLC-QqQ_LIT_-MS/MS. The findings will enhance further in depth biological studies of the endophytic actinobacterial population for sustainable development of agricultural and pharmaceutical pursuits.

## Results

### Recovery of endophytic actinobacteria

In total, one hundred and sixty-nine endophytic actinobacterial isolates were obtained from root, stem and leaf parts of *R*. *ellipticum* based on colony morphology and formation of color by aerial and substrate mycelia. Out of 169 isolates, the maximum number of isolates were obtained from root (n = 79; 46.7%) followed by leaf (n = 48; 28.4%) and stem (n = 42; 24.8%) selected using five nutritional media. Most of them were slow growers and formed different colors including brownish white, orange, blackish white, etc. The Scanning electron microscope (SEM) result showed the aerial mycelia with spiral chain morphology (Supplementary Fig. S1).

### Evaluation of antimicrobial activity

All endophytic isolates were evaluated for their antimicrobial potential against four bacterial pathogens (*Staphylococcus aureus*, *Bacillus subtilis*, *Pseudomonas aeruginosa*, and *Escherichia coli*), three fungal pathogens (*F*. *proliferatum*, *F*. *oxy* f. sp. *ciceri* and *F*. *oxysporum*) and a yeast pathogen *C*. *albicans*. Out of 169 isolates, 81 isolates (47.9%) showed positive activity against 4 out of the 8 tested pathogens and all the isolates exhibited a positive result against *E*. *coli*. However, four isolates showed significant antimicrobial activity against more than six tested pathogens. Isolate BPSAC77 exhibited maximum antimicrobial potential against *S*. *aureus* (12.6 mm) and *P*. *aeruginosa* (10.4 mm) whereas, isolates BPSAC147, BPSAC121 and BPSAC101 showed significant antibacterial activity against *E*. *coli* (13.2 mm), *C*. *albicans* (9.8 mm) and *B*. *subtilis* (11.6 mm) respectively (Supplementary Table S1). Among all isolates tested isolate BPSAC77 showed significant antibacterial activity against all tested bacterial pathogens.

Out of 81 isolates, 72 isolates showed considerable growth inhibitory activity against three fungal phytopathogens i.e. *F*. *proliferatum*, *F*. *oxy* f. sp.*ciceri* and *F*. *oxysporum* with the percentage of inhibition ranging from 32% to 72%. Among them, Isolate BPSAC77 displayed the highest percentage of inhibition against *F*. *proliferatum* (72%) and *F*. *oxy* f. sp. *ciceri* (54%) whereas; isolate BPSAC121 showed maximum antagonistic activity against *F*. *oxysporum* (63%). (Supplementary Table S1).

### Identification by amplification of 16S rRNA gene and phylogenetic analysis

**Figure 1:**
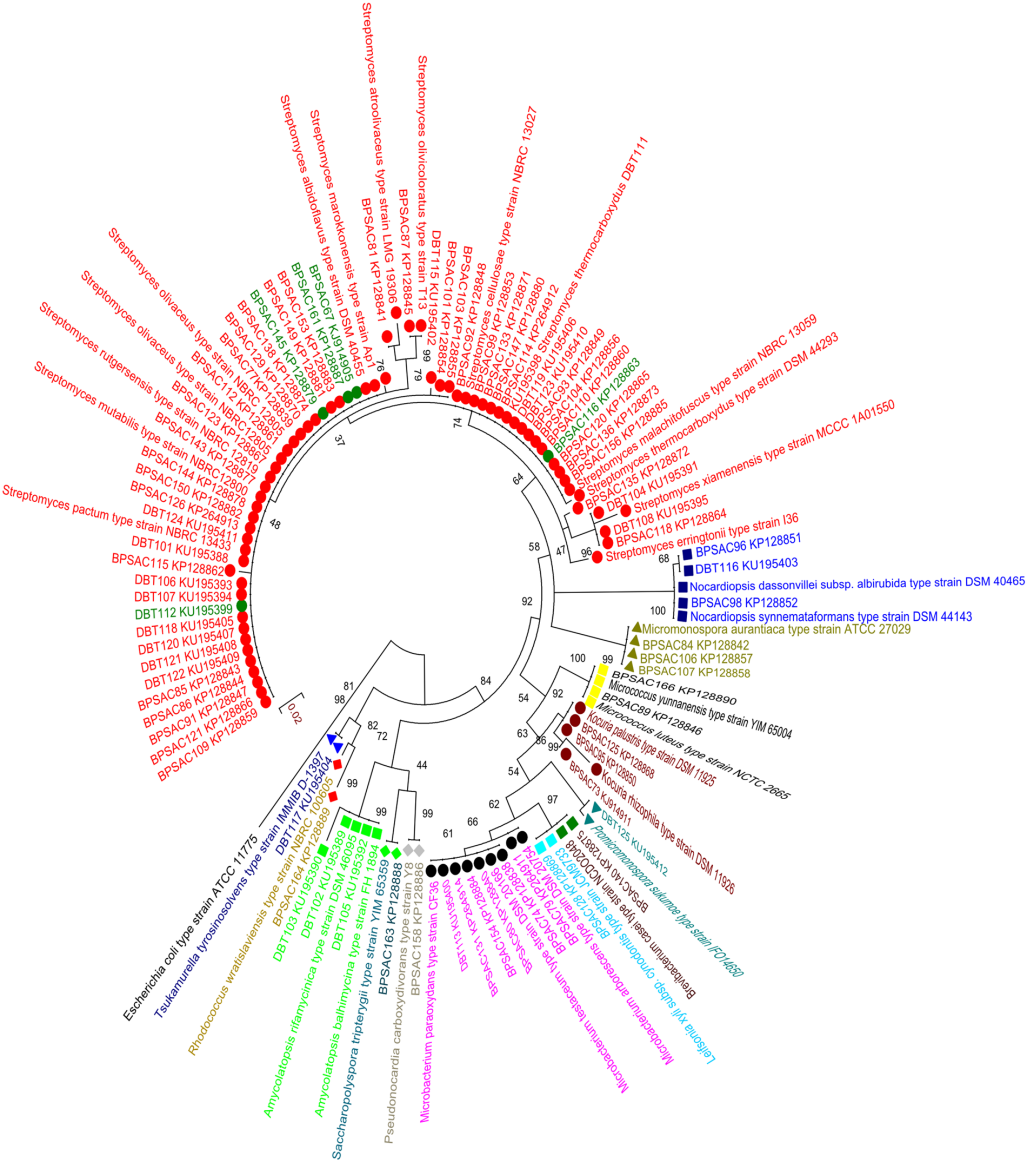
Maximum likelihood phylogenetic tree using Kimura-2 parameter model based on 16S rRNA gene sequences of endophytic actinobacterial isolates showing the phylogenetic relationship between the endophytes sequences with closest type strain sequences. Numbers at branches indicate bootstrap values in 1,000 replicates.

The obtained isolates that exhibited potent antimicrobial activity were identified using the 16S rRNA region amplification. The obtained16S rRNA gene sequences were aligned using BLAST analysis with the reference strains downloaded from Eztaxon database. Analysis of partial 16S rRNA gene sequences (820–980 bp) of 81 potential strains exhibited a high level of sequence similarity (97–99%) with the existing strains in the database (Supplementary Table S2). The strains were divided into 12 families and 15 genera. Most of the isolates were grouped into *Streptomyces* (n = 49; 60.49%), followed by *Microbacterium* (n = 6; 7.4%), *Actinomycete* (n = 5; 6.17%), *Micromonospora* (n = 3; 3.7%), *Kocuria* (n = 3; 3.7%), *Nocardiopsis* (n = 3; 3.7%), *Amycolatopsis* (n = 3; 3.7%), *Micrococcus*(n = 2; 2.46%), one each were sorted as *Brevibacterium*, *Pseudonocardia*, *Leifsonia*, *Rhodococcus*, *Tsukamurella*, *Promicromonospora* and *Saccharopolyspora* (n = 1; 1.23%). Sequence analysis confirmed that 49 isolates belonging to *Streptomyces* showed 98–100% identity followed by 5 isolates (BPSAC67, BPSAC116, BPSAC145, BPSAC161 and DBT112) that showed 98–100% identity with the sequences retrieved from the genus *Actinomycete*. The phylogenetic tree was constructed based on maximum-likelihood methods with Kimura 2-parameter model using Mega 5.05. The estimated Transition/Transversion bias (R) is 1.54. The phylogenetic tree demonstrate that all the endophytic actinomycetes strains classified into three different clades (clade I, clade II and clade III) and is supported by a high bootstrap value (84%). Most of the genus *Streptomyces* forms a major (clade I), along with most closely related type strains recovered from GenBank databases with the exception of *Actinomycete*, also falls in the same clade under a bootstrap support value of 74%. All of the sequences within clade II and clade III are closely related with their type strains under bootstrap values of 58% and 98% respectively. All the 16S rRNA gene sequences have been deposited in NCBI database with accession numbers KP128838-KP128890; KP264911-KP264914; KJ914911; KJ914905; KU195388-KU195412 (Fig. 1).

### Plant growth promoting traits of antagonistic endophytic actinobacteria

#### Phosphate solubilization

Out of 81 isolates, 21 (25.9%) strains showed significant phosphate solubilizing ability under *in vitro* conditions by producing prominent clear halo zones around colonies grown on Pikovskay’s agar media. The isolate *Streptomyces olivaceus* strain BPSAC77 showed the highest phosphate solubilization index (9.7 mm) followed by *Streptomyces* sp. strain BPSAC101 (8.4 mm), *Streptomyces* sp. strain BPSAC121 (8.2 mm) and *Streptomyces thermocarboxydus* strain BPSAC147 (7.6 mm) (Supplementary Table S3).

#### Indole acetic acid (IAA) and Ammonia production

Out of 81 strains, 36 strains (44.4%) were positive for indole acetic acid (IAA) production, among them 23 isolates belong to genus *Streptomyces* and some isolates belong to the genus *Actinomycetes*, *Microbacterium*, *Micromonospora*, *Leifsonia*, *Brevibacterium*, *Pseudonocardia*, *Promicromonospora*, *Kocuria* and *Amycolatopsis*. Quantitative estimation of all positive isolates was performed in ISP1 broth medium with the addition of tryptophan and production ranged from 7.4 to 52.3 µg/ml. The maximum IAA yield was observed in *Streptomyces olivaceus* strain BPSAC77 (52.3 µg/ml) followed by *Streptomyces* sp. strain BPSAC121 (47.8 µg/ml), *Streptomyces* sp. strain BPSAC101 (42.6 µg/ml) and *Streptomyces thermocarboxydus* strain BPSAC147 (39.4 µg/ml) (Supplementary Table S3).

Out of 81 strains, 47 strains (58.02%) were positive for ammonia production. Quantitative estimation of ammonia production by all positive isolates in peptone water broth ranging from 8.6 to 82.3 mg/ml. Isolate BPSAC121 produced the maximum amount of ammonia (82.3 mg/ml) followed by BPSAC77 (78.8 mg/ml) and BPSAC101 (74.2 mg/ml) (Supplementary Table S3).

#### Cellulase and Amylase production assays

Out of 81 isolates, 24 isolates (29.6%) showed cellulase production by the formation of clear halo zones surrounding the colonies. Among all the positive isolates, BPSAC77 showed maximum cellulase production (74.2 U/ml) followed by BPSAC101 (67.5 U/ml), BPSAC121 (59.7 U/ml) and BPSAC147 (54.6 U/ml) (Supplementary Table S3).

For amylase screening, 39 isolates (48.1%) were positive for amylase production with quantitative estimation ranging from 76.4 to 32.6 U/ml. Isolate BPSAC147 showed the highest amylase production (76.4 U/ml) followed by BPSAC77, BPSAC101 and BPSAC121 with 74.8 U/ml, 54.4 U/ml and 51.3 U/ml respectively (Supplementary Table S3).

#### Antibiotic sensitivity assay

All endophytic actinobacteria strains were screened for antibiotic sensitivity profiling against 12 standard antibiotics *viz*. penicillin G (P), tetracycline (T), nalidixic acid (Na), ampicillin (A), vancomycin (V), gentamicin (G), norfloxcin (N), chloramphenicol (C), erythromycin (E), nystatin (Ny), rifampicin (R) and streptomycin (S). All isolates exhibited resistance to penicillin G (P) and ampicillin (A) (100% each) followed by Neomycin (n = 54; 66.6%), nystatin (n = 48; 59.2%) and streptomycin (n = 42; 51.8%) whereas, most of the strains reveal high sensitivity to tetracycline and chloramphenicol (100% each) followed by erythromycin (n = 73; 90.1%), norfloxcin (n = 16; 79.01%) and vancomycin (n = 52, 64.1%). The isolates *Streptomyces olivaceus* strain BPSAC77, *Streptomyces* sp. strain BPSAC101, *Streptomyces* sp. strain BPSAC121 and *Streptomyces thermocarboxydus* strain BPSAC147 showed resistance against 7 out of 12 antibiotics (Supplementary Table S3).

### Modular polyketide synthases (PKSI, PKSII) and nonribosomal peptide synthetase (NRPS) gene clusters

Using degenerate primers, PKSI, PKSII and NRPS genes were successfully amplified from selected isolates of endophytic actinobacteria. The result exhibited that the PKSI gene fragment was detected in 25 isolates, whereas, the PKSII gene fragment was detected in 41 isolates. Also, the NRPS gene was detected in 32 isolates. Out of 81 isolates, 17 Strains showed positive amplification for all three genes. All the sequences were deposited to the NCBI GenBank and assigned accession numbers: PKSI gene (KU956018 to KU956041); PKSII gene (KU879295 to KU879329 & KT87712 to KT87717) and NRPS gene (KU899056 to KU899086). PKSI, PKSII and NRPS gene sequences were deposited for the first time in NCBI GenBank database of genus *Microbacterium*, *Nocardiopsis*, *Leifsonia*, *Actinomycete*, *Micromonospora*, *Pseudonocardia*, *Brevibacterium*, *Saccharopolyspora*, *Kocuria*, *Rhodococcus* and *Micrococcus* (Supplementary Table S2).

### Expression of antimicrobial biosynthetic genes (PKSI and NRPS) using *Real Time-qPCR*

#### Relative quantification of PKS type I and NRPS gene

**Figure 2:**
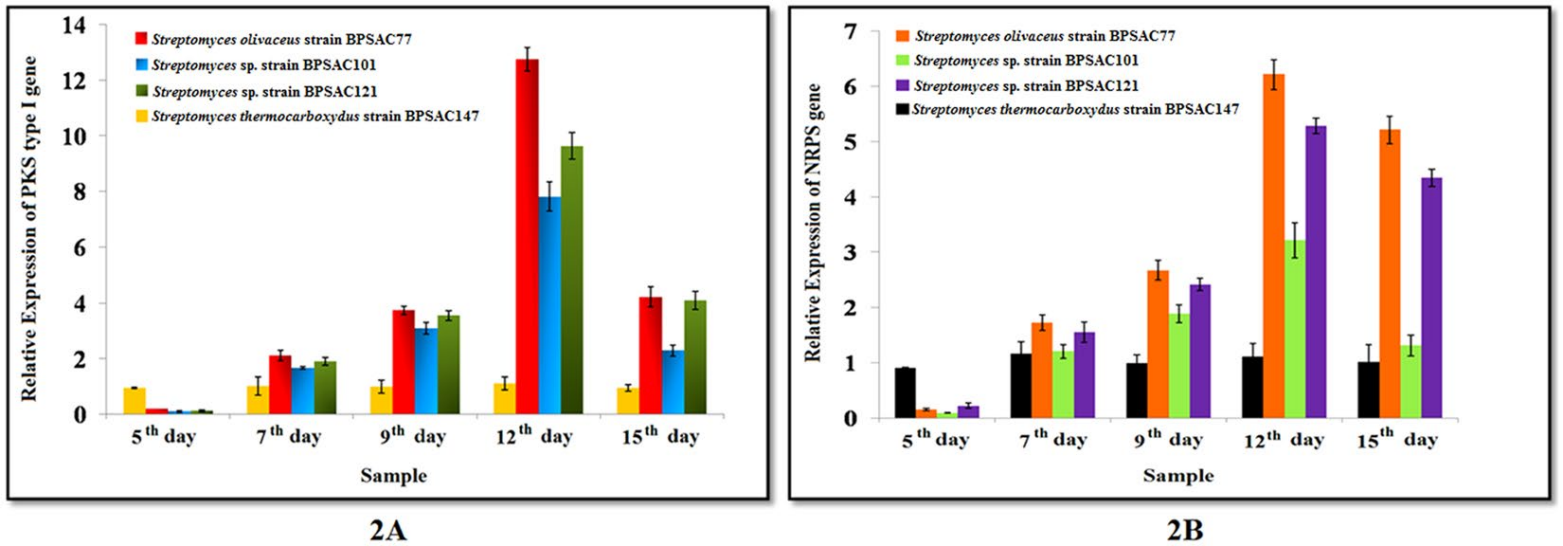
Relative gene expression of PKS type I gene and NRPS gene on five alternate days across strains BPSAC77, BPSAC101, BPSAC121 and BPSAC147. The expression level calculated by the formula 2^−ΔΔCt^ represents the x-fold difference from the calibrator and 16S rRNA gene as endogenous control. Threshold cycle (C_T_) was compared with log_10_ relative copy number of the sample from a dilution series. Error bars indicate standard error. Sample after 12^th^ day of incubation shows highest expression level.

Four strains BPSAC77, BPSAC101, BPSAC121 and BPSAC147 were selected based on their antimicrobial potential against bacterial pathogens. The mRNA transcripts of PKS-I and NRPS were detected from the bacterial strains at selected intervals from 5 to 15 days. cDNAs of BPSAC147 was taken as reference throughout the experiment. A gradual increase in PKSI transcript copy number was noticed in all the bacterial strain from the 5 to the 12 day. A sharp decrease in expression was observed on the 15 day of inoculation. BPSAC77 showed highest expression level with almost a 13-fold increase detected on the 12^th^ day of inoculation (Fig. 2A). An almost similar trend was noticed in the NRPS gene expression pattern. A gradual increase in expression level was noticed from the 5day to the 12 day of inoculation; however mRNA expression was almost stable in BPSAC77 on the 15 day of inoculation too. A 6-fold increase in expression was observed in BPSAC77 on the 12 day of inoculation (Fig. 2B). Furthermore, both targets showed highest relative expression in BPSAC77 at different time intervals.

#### Antioxidant activity

**Figure 3:**
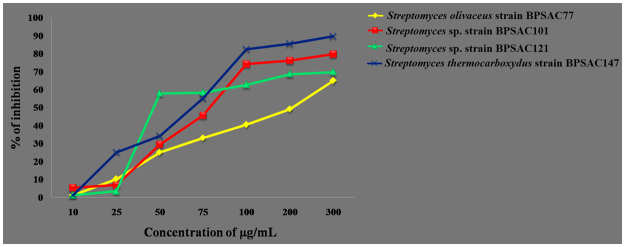
Scavenging activity (%) on DPPH radicals (IC_50_) of methanolic extract of potential four endophytic actinobacterial strain (*Streptomyces olivaceus* strain BPSAC77; *Streptomyces* sp. strain BPSAC101; *Streptomyces* sp. strain BPSAC121 & *Streptomyces thermocarboxydus* strain BPSAC147) at different concentration (µg/mL). Ascorbic acid was used as a positive control in DPPH radical scavenging test and the experiment was performed in triplicate for all the samples.

In the present study, the four potential extracts (BPSAC77, 101, 121 and 147) were evaluated for their DPPH scavenging activity. It was shown that all four strains showed effective DPPH radical scavenging activity, but the extract from strain BPSAC77 showed the maximum significant scavenging activity (IC_50_ value of 43.2 µg/mL) (Fig. 3). This is first report of antioxidant activity in *Streptomyces olivaceus* strain BPSAC77 and *Streptomyces thermocarboxydus* sp. strain BPSAC147 (IC_50_ values of 75.4 µg/mL). These results demonstrate that lower concentrations of endophytic actinobacteria ethyl acetate extract were successful in scavenging the DPPH radical, which indicates a very good antioxidant property.

### Detection and quantification of bioactive compounds by UHPLC-QqQ_LIT_-MS/MS

#### Validation of Method

The proposed UPLC-MRM method for quantitative analysis was validated as per the procedure of the international conference on harmonization (ICH, Q2R1) by linearity, LOQs and LODs, precision, solution stability, and recovery.

#### Linearity, limits of detection (LOD) and quantification (LOQ)

A series of concentrations of standard solution were prepared to construct calibration curves. The peak areas were plotted against the corresponding concentrations to obtain the calibration curves. LOD and LOQ were determined by comparison to the calibration curve. LOD and LOQ were determined as. LOD = (3.3 × Sy.x)/S; LOQ = (10 × Sy.x)/S (Where, Sy.x is the standard deviation of residuals from the line; S is the slope). The results were listed in Supplementary Table S4. The calibration curves showed a good linearity with correlation coefficient (r^2^) ranges from 0.9989 to 0.9999. The LOD and LOQ for each reference analyte were less than 0.52 ng/mL and 0.78 ng/ml, respectively.

#### Precision, Stability and Recovery

Precision was measured by relative standard deviation (RSD) with intra-day and inter-day variations evaluated by determining six replicates of the analytes on a single day and the experiments were repeated on three successive days. The intra-day and inter-day precision was found to be less than 2.01%. Stability of the samples was analyzed by replicate injections at 0, 2, 4, 8, 12 and 24 h. The stability RSD% value of the analytes is ≤2.45%. To evaluate accuracy, a recovery test was applied by spiking three different concentration levels (high, middle and low) of the standards analytes into the samples. The experiment was performed in triplicate. The analytical method used showed a good accuracy with overall recovery in the range from 97.98–102.12% (RSD ≤ 1.45%) for all analytes (Supplementary Table S4).

#### Quantitative analysis

**Figure 4:**
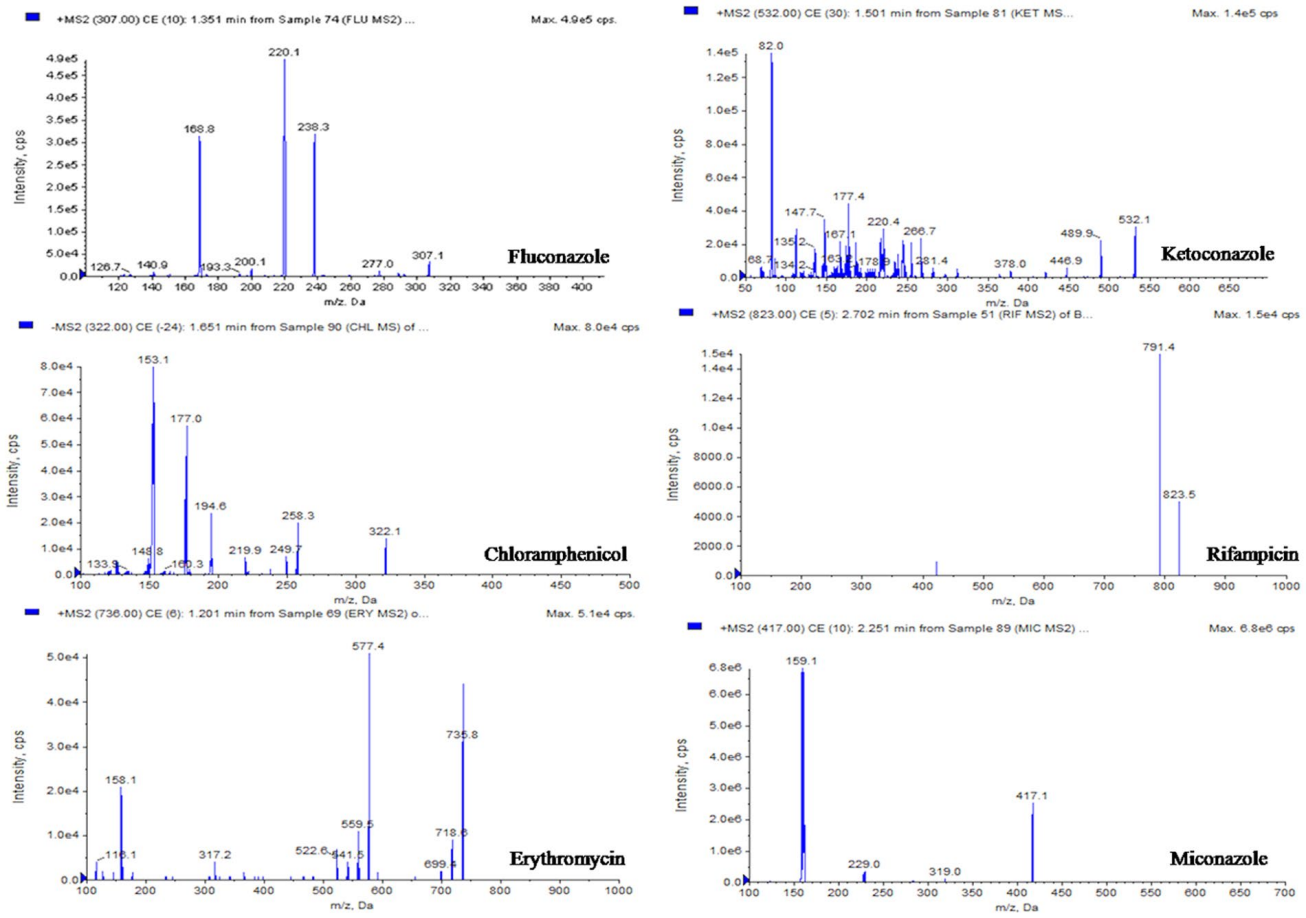
MS/MS spectra of reference analytes of standard six antibiotics; Fluconazole, Chloramphenicol, Erythromycin, Ketoconazole, Rifampicin, Miconazole.

**Figure 5:**
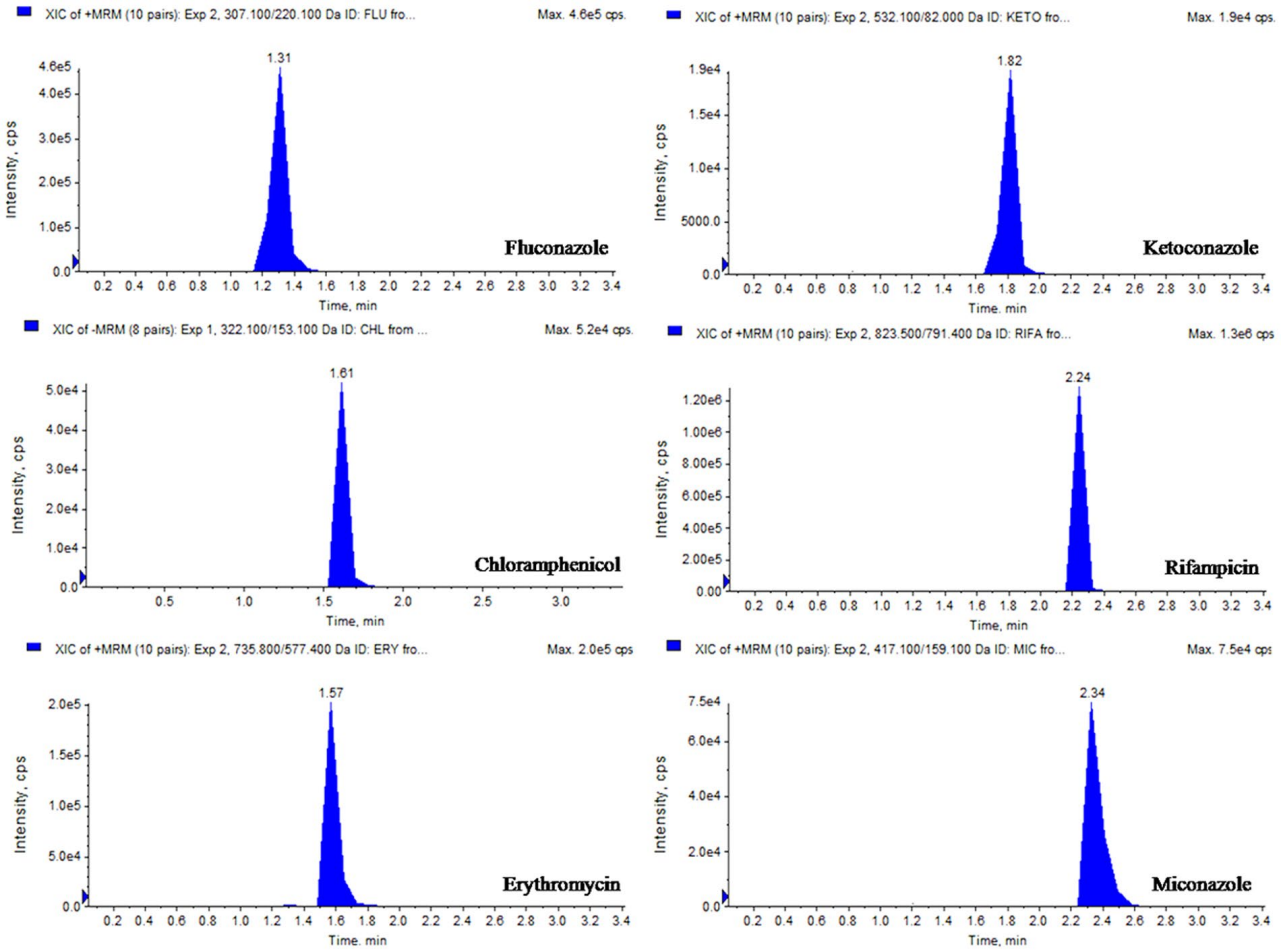
MRM-extracted ion chromatogram (XICs) of six pair of antibiotics obtained by UPLC-ESI–MS/MS.

**Figure 6:**
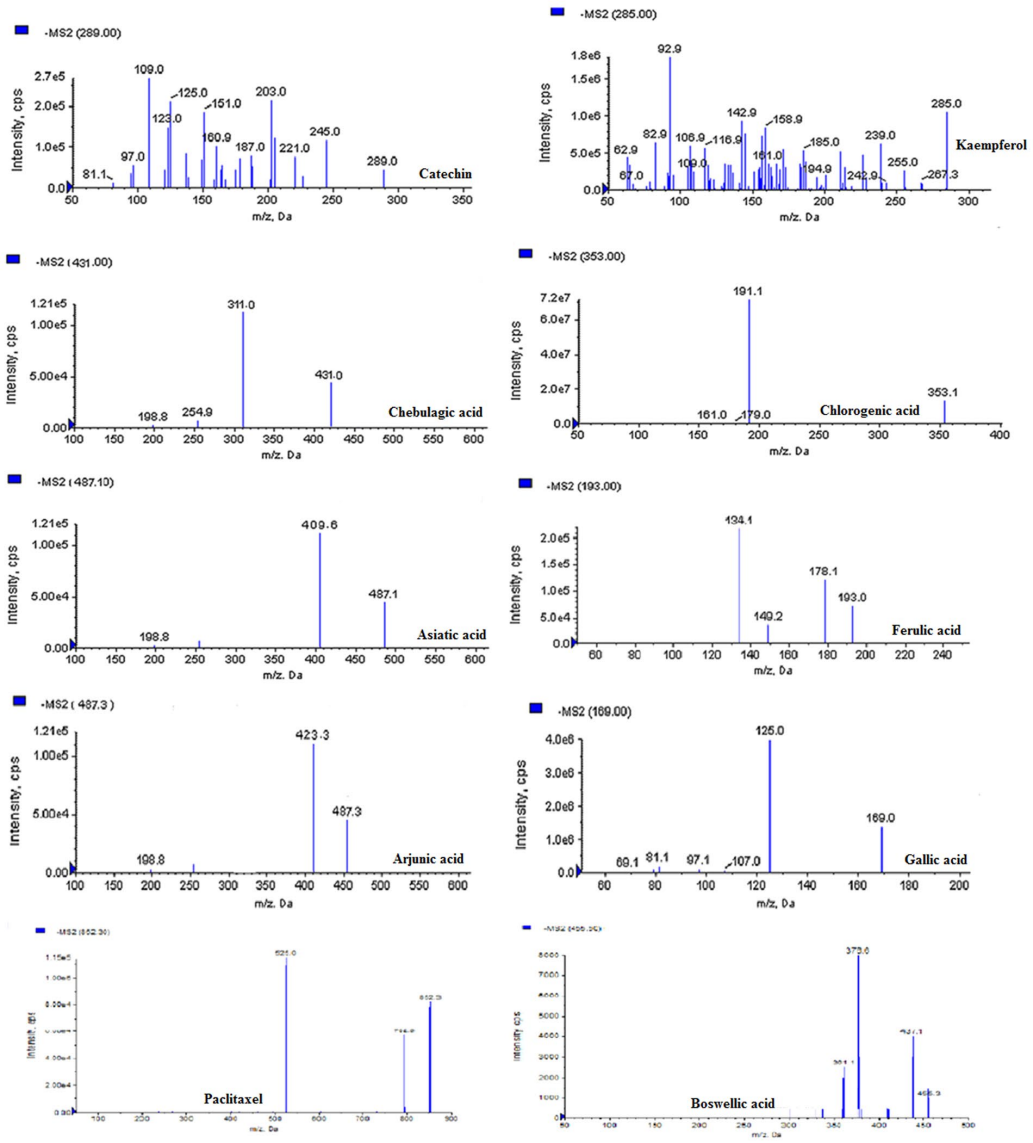
MS/MS spectra of reference analytes of standard nine phenolic compound; Catechin, Kaempferol, Chebulagic acid, Chlorogenic acid, Asiatic acid, Ferulic acid, Arjunic acid, Gallic acid, Boswellic acid and an anticancerous compound Paclitaxel.

**Figure 7:**
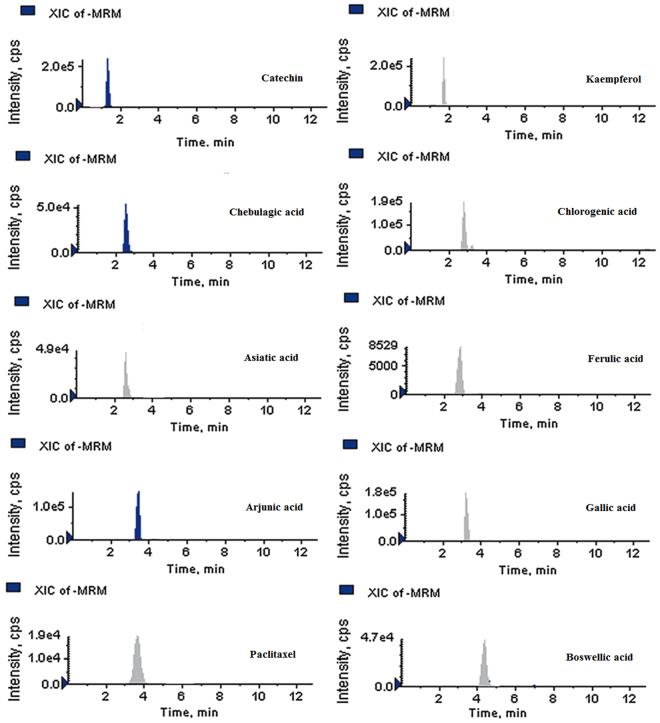
MRM-extracted ion chromatogram (XICs) of nine pair of phenolic and an anticancer compound obtained by UPLC-ESI–MS/MS.

In this study, the UHPLC-QqQ_LIT_-MS/MS method was applied to detect and quantify sixteen bioactive compounds that includes six antibiotics (fluconazole, chloramphenicol, erythromycin, ketoconazole, rifampicin and miconazole), eight phenolic compounds (catechin, kaempferol, chebulagic acid, chlorogenic acid, ferulic acid, arjunic acid, gallic acid and boswellic acid) and Paclitaxel, an anticancer compound. Among the detected antibiotics, three were antifungal (ketoconazol, fluconazole and miconazole) and other three were antibacterial (chloramphenicol, erythromycin and rifampicin). All six antibiotics were detected and quantified in the ethyl acetate extract of *Streptomyces olivaceus* strain BPSAC77 and *Streptomyces* sp. strain BPSAC121. *Streptomyces* sp. strain BPSAC101 and *Streptomyces thermocarboxydus* strain BPSAC147 showed the presence of an antifungal antibiotic, ketoconazol, at a concentration of 276 μg/g and 289 μg/g respectively, which was the first report for this antibiotic from endophytic actinobacterial strains along with fluconazole, rifampicin and miconazole (Table 1). Gallic acid was detected in all the selected strains (BPSAC77, BPSAC101, BPSAC121 and BPSAC147). A natural phenolic ferulic acid which has direct antitumor activity against breast cancer and liver cancer was detected and quantified in *Streptomyces* sp. strain BPSAC 121. Additionally, paclitaxel an anticancer compound used to treat various types of cancer was detected at the highest concentration in *Streptomyces olivaceus* strain BPSAC77 (23.4 µg/g) followed by *Streptomyces* sp. strain BPSAC101 (18.2 µg/g) and *Streptomyces* sp. strain BPSAC121 (10.2 µg/g). Furthermore, the chebulagic acid, a phenolic compound against *Staphylococcus aureus* and *Candida albicans* was detected in *Streptomyces olivaceus* strain BPSAC77 and *Streptomyces* sp. strain BPSAC121 which may play a role in the antimicrobial potential of strain BPSAC77 and BPSAC121 as found in the antimicrobial results. This is first report of the occurrence of chebulagic acid, the benzopyran tannin antioxidant in an endophytic actinobacterial strain isolated from a medicinal plant (Table 1). The MS spectra and MRM extracted ion chromatogram of 16 mixed standards and the selected four strains are shown in Figs 4, 5, 6 and 7 respectively.

**Table 1:** Content of bioactive compounds (µg/g) detected and quantified in selected four endophytic actinobacterial strains.

SL. No	BPSAC77	BPSAC101	BPSAC121	BPSAC147
Fluconazole	7.13	—	1.32	—
Chloramphenicol	75.3	—	11.8	—
Erythromycin	19.5	—	0.32	—
Ketoconazole	231	276	110	289
Rifampicin	36.3	—	36.6	—
Miconazole	226.3	—	233.6	—
Catechin	18.8	—	14.3	—
Kaempferol	22.1	32.1	—	—
Chebulagic acid	2.7	—	9.7	2.6
Chlorogenic acid	5.0	23.2	18.9	—
Asiatic acid	—	5.4	—	11.7
Ferulic acid	—	—	2.8	—
Arjunic acid	12.5	20.4	—	15.6
Gallic acid	92.0	48.3	26.5	18.0
Paclitaxel	23.4	18.2	10.2	—
Boswellic acid	—	12.0	8.9	—

*Not detected.

## Discussion

Endophytic microorganisms like endophytic actinobacteria inhabit within the plant tissues acquired certain unique characteristics that enable them to flourish without any obvious infectious symptoms to the host. They are recognized for their capacity to produce naturally occurring bioactive molecules^15,23,36^. Endophytic actinobacteria obtained from medicinal plants are of prime importance nowadays with the scope for the exploration for novel natural products gaining more strength^37,38^. These findings encouraged us to study the functionality of endophytic actinobacteria with a main focus to understand their biosynthetic potential and the production of bioactive compounds associated with the traditionally used medicinal plant *R*. *ellipticum* in Mizoram, northeast India.

In total 169 isolates were obtained from different tissues (leaf, stem and root) of *R*. *ellipticum* collected from Dampa tiger reserve forest and Murlen National Park of Mizoram, India using five minimal nutrient media. Out of 169 isolates, genus *Streptomyces* was found highest (n = 118, 69.8%), similar findings from different hosts have been previously reported^32,38^. This study also identified some rare genera obtained from the endospheric atmosphere such as *Microbacterium*, *Amycolatopsis*, *Tsukamurella*, *Nocardiopsis*, *Actinomycete*, *Promicromonospora*, *Leifsonia*, *Micromonospora*, *Pseudonocardia*, *Brevibacterium*, *Saccharopolyspora*, *Kocuria*, *Rhodococcus* and *Micrococcus* were reported for the first time from *R*. *ellipticum*. These findings were in concurrence with the reports of several researchers who reported uncommon genera from other medicinal plants like *Leifsonia* & *Microbacterium* from *Clerodendrum colebrookianum*^32^; *Amycolatopsis* from *Osyris wightiana*^38^; *Tsukamurella* and *Nocardiopsis* from *Maytenus austroyunnanesis*^39^, *Actinomycete* from *Mirabilis jalapa*^32^ and *Promicromonospora* from *Cymbopogon citrates*^39^ which proved their endophytic nature.

The population of endophytic actinobacteria was determined to be greater in root tissues (n = 79; 46.7%) followed by stem (n = 42; 24.8%) and leaf (n = 48; 28.4%), which reveals that actinobacteria are more prevalent in the root tissues of the host plant. Several researchers have reported similar results where the greatest number of endophytic actinobacteria was recovered from root tissues^40,41^.

All the isolates were checked for their antimicrobial activity against Gram-positive and Gram-negative bacteria and *C*. *albicans*. Previous studies revealed that the endophytic actinobacterial isolated from several medicinal plants possess prominent antimicrobial potential against bacterial and fungal pathogens^39^. In our study, 81 out of 169 isolates showed positive *in vitro* antimicrobial activity against four tested pathogens. For example, isolate BPSAC77 exhibited the highest antimicrobial activity against *S*. *aureus* (12.6 mm) and *P*. *aeruginosa* (10.4 mm) whereas isolates BPSAC147, BPSAC121 and BPSAC101 revealed potential antimicrobial activity against *E*. *coli* (13.2 mm), *C*. *albicans* (9.8 mm) and *B*. *subtilis* (11.6 mm) which was in concurrence with the findings of Wu *et al*.^42^ who stated that *Streptomyces* strain SAUK6233 isolated from *Mosla dianthera maxima* plant showed the highest antibacterial activity against all tested indicator organisms. Similarly, Passari *et al*.^43^ also reported that endophytic bacteria from *Clerodendrum colebrookianum* Walp. has anticancer, antioxidant, plant growth promoting and antimicrobial activities.

Among all, antagonistic activity was shown by 72 isolates (42.6%) against three fungal phytopathogens with the percentage of inhibition ranging from 32 to 72%. This finding is in consensus with Verma *et al*.^19^ who reported 60% of the endophytic actinobacteria isolates obtained from *Azadirachta indica* showed wide-spectrum antagonistic potential. Six isolates BPSAC (77, 101, 121, 147 and 164) and DBT115 showed antagonistic activity against all the selected phytopathogens. Among the phylum actinobacteria, the genus *Streptomyces* has an outstanding record for the production of bioactive metabolites and natural antibiotics^44,45^. The antagonistic potential of endophytic actinobacteria observed in this study is in accordance with findings previously reported by Verma *et al*.^19^ and Graner *et al*.^46^.

The isolates that showed significant antimicrobial activities were identified using 16S rRNA gene amplification and a phylogenetic tree was constructed to assign them a unique taxonomic unit. The isolates were classified into just fifteen genera which indicate a strong relationship of endophytic actinobacteria with medicinal plants. *Streptomycetaceae* was the dominant family followed by *Pseudonocardiaceae*, *Microbacteriaceae*, *Micrococcaceae*, *Micromonosporaceae*, *Nocardiopsaceae*, *Promicromonosporaceae*, *Actinomycetaceae*, *Nocardiaceae*, *Brevibacteriaceae*, *Nocardiopsaceae* and *Tsukamurellaceae*. The phylogenetic tree of the isolates constructed in this study was more diverse in comparison to the endophytic actinobacteria reported from other medicinal plants and the rain forest of China^18,19^. All the *Streptomyces* fall in the same clade except for *Actinomycete* which clustered together^47^. On the other hand, rare genera like *Amycolatopsis*, *Tsukamurella*, *Promicromonospora*, and *Nocardiopsis* formed a separate cluster with their type strains. The findings are in concurrence with Nimnoi *et al*.^16^ who stated that the isolate S4301 and S4201 were closely clustered to the type strains of *Nocardia alba* DSM44684 and *Pseudonocardia halophobica* DSM433089 under a bootstrap supported value of 62% and 88% respectively. To the best of our knowledge and extensive literature search, this is the first attempt towards the isolation and characterization of endophytic actinobacteria from *R*. *ellipticum*, though; Qin *et al*.^39^ isolated *Amycolatopsis* from the roots of the medicinal plant *Osyris wightiana*; *Tsukamurella* was isolated from the stem of *Maytenus austroyunnanensis*; and *Promicromonospora* was isolated from the root of *Cymbopogon citratus*.

Phosphorus is among one of the most important nutrients required for plant growth and development. In soil it is mostly available as insoluble phosphorous and becomes a limiting factor for plant growth^48^. Several researchers have stated that phosphate-solubilizing bacteria can be used in crop fields to enhance plant growth by increasing organic phosphorus uptake^49,50^. In the present study, 21 out of 81 strains revealed a clear halo zone surrounding the colonies indicating their phosphate solubilization effectiveness which ranged from 3.8 mm to 9.7 mm. Among them, isolate *Streptomyces olivaceus* strain BPSAC77 showed the highest phosphate solubilization efficiency (9.7 mm) followed by *Streptomyces* sp. strain BPS101 (8.4 mm), *Streptomyces* sp. strain BPSAC121 (8.2 mm) and *Streptomyces thermocarboxydus* strain BPSAC147 (7.6 mm). This is in concurrence with Hamdali *et al*.^51^ and Passari *et al*.^52^ who mentioned that most of the *Streptomyces* isolates show phosphate solubilizing activity. Bashan *et al*.^49^ noted the capacity of phosphate solubilizing microorganisms to solubilize inorganic aluminum phosphate (AlPO_4_.2H_2_O) and iron phosphate (FeSO_4_.2H_2_O) both which are widely available in agricultural soils and are very stable minerals.

Phytohormone plays a significant role in plant growth promotion. Ribeiro *et al*.^53^ suggested that IAA production helps the plants to grow root parts, which increases the plants nutrient uptake. In our present study, out of 81 isolates, 36 strains (44.4%) were found to be positive for the production of indole acetic acid (IAA) with production ranging from 7.4 to 52.3 µg/ml in the presence of L-tryptophan. This finding is in consensus with Khamna *et al*.^54^ who stated that IAA production levels ranged from 13.73 to 142 µg/ml. The variation in the ability of PGPR to produce IAA found in the present study had also been reported earlier^55^. This variation may be due to the location of the genes involved, regulatory sequences, as well as the availability of enzymes that can modify active free IAA^56^. Out of 36 isolates, the IAA biosynthetic gene was detected in 23 isolates. This result is like findings reported by Passari *et al*.^52^ who stated all plant growth promoting endophytic actinobacterial strains having the IAA encoding gene.

Ammonia production is an indirect mechanism of plant growth promotion and can play a significant role in suppressing phytopathogens^57^. In the present study, all isolates showed ammonia production in peptone water broth with levels ranges from 8.6 to 82.3 mg/ml. Qualitative estimation showed that *Streptomyces* sp. strain BPSAC121 produced the greatest amount of ammonia (82.3 mg/ml). The findings were supported by similar results by Nimnoi *et al*.^16^ who stated that isolate *Streptomyces hainanensis* strain S4303 obtained from *Aquilaria crassna Pierre* ex Lec showed ammonia production of 60.0 mg/ml. Marques *et al*.^58^ also proposed that ammonia production by bacteria can both accumulate and provide N_2_ to the plant promoting shoot and root elongation and therefore increasing plant biomass.

Microorganisms with cellulase and amylase activity do not merely help in organic matter decomposition and plant growth promotion, but also play a critical role in disease suppression by inhibiting soil borne pathogens^59^. Cellulase is an important industrial enzyme used in depolymerization of cellulose into fermentable sugar^60^. In our study, out of 81 strains, 24 strains (29.6%) formed a clear zone around colonies on CMCase agar media. This result was like those reported by Sirisha *et al*.^61^ who stated that 24% of isolates produce cellulase with levels ranging from 0.40 to 0.75 IU/ml. Among the positive strains, *Streptomyces olivaceus* strain BPSAC77 produced the greatest amount of cellulase (74.2 U/ml). These findings are similar with Jang and Chen^62^, who found that *Streptomyces transformant* T3-1 showed a cellulase production of 2.6 U/ml. Amylases are a class of industrial enzymes which account for approximately 25–30% of the world enzyme market^63,64^. They are widely found in microbial, plant and animal kingdoms. In our study, out of 81 isolates, 39 isolates (48.1%) were positive for amylase production with levels ranging from 76.4 to 32.6 U/ml. Similar findings were reported by Sirisha *et al*.^61^ who stated that 81% of isolates had the ability to produce amylase. The maximum amylase activity was displayed by the *Streptomyces thermocarboxydus* strain BPSAC147 (76.4 U/ml). This finding agrees with our earlier reports Passari *et al*.^43^.

81 selected isolates were tested for their antibiotic sensitivity against twelve standard antibiotics. Among them, four strains: *Streptomyces olivaceus* strain BPSAC77, *Streptomyces* sp. strain BPSAC101, *Streptomyces* sp. strain BPSAC121 and *Streptomyces thermocarboxydus* strain BPSAC147 exhibited resistance against seven out of the 12 tested antibiotics. This finding was reported previously by Passari *et al*.^32^. Additionally, endophytic actinobacteria i.e. *Micrococcus* obtained from *Andrographis paniculata* leaves have antibiotic resistance as described by Pal *et al*.^65^. All the isolates were resistant to ampicillin. These results are also supported by the findings of Falcao *et al*.^66^ and Reboucas *et al*.^67^.

Biosynthetic potential of endophytic strains including actinobacterial strains was evaluated by PCR based detection of secondary metabolic biosynthetic genes like PKS type I, PKS type II and NRPS^29,31^. At the same time, the isolates with antimicrobial properties do not compare with the percentage of isolates with positive detection of PKS (type I & type II) and NRPS genes^39^. In the present study, PKS type I, PKS type II and NRPS genes were detected in 25, 41 and 32 isolates respectively, which also showed significant antimicrobial activities along with PGPR activity. This is contrary to the results of Qin *et al*.^39^ and Passari *et al*.^32^ who stated that there is no direct relationship between antimicrobial activity against pathogens and detection of functional genes. Biosynthetic genes sequences (PKSI, PKSII and NRPS) were deposited for the first time to NCBI GenBank database for genus *Microbacterium*, *Nocardiopsis*, *Leifsonia*, *Actinomycete*, *Micromonospora*, *Pseudonocardia*, *Brevibacterium*, *Saccharopolyspora*, *Kocuria*, *Rhodococcus* and *Micrococcus*.

Biosynthetic gene PKS type I and NRPS showed that the highest relative expression and greatest variation was observed in all selected strains on the 12^th^ day. After that, relative expression was drastically decreased. Gohain *et al*.^20^ have reported that the endophytic actinomycetes associated with medicinal plants demonstrated PKS type II gene expression was up-regulated 12-fold on the 7^th^ day of incubation for *Streptomyces antibioticus* (EAAG90). The findings suggested that all the investigated strains produce elevated amounts of bioactive compound on the 12 day of incubation.

DPPH radical scavenging activity has been extensively used to determine antioxidant potential of various organisms^68^. The ability of antioxidants to scavenge DPPH radicals is due to their hydrogen donating ability becoming stable diamagnetic molecules. The compounds reduce the purple colored DPPH radical to a yellow colored compound and the degree of color variation is like the extent of the hydrogen donating ability of the tested sample^69^. Even though the radical scavenging activity of the tested extracts was less than that of ascorbic acid, it was obvious that the extracts showed hydrogen donating ability and can serve as free radical scavengers^69^. The result supports earlier reports, which also reveal moderate scavenging activity of actinobacteria extracts^70^. Some recent studies have revealed that free oxygen plays a vital role for causing several diseases such as cancer, autoimmune disorders and cardiovascular and neurodegenerative diseases^71,72^. This demands further investigation of novel and potent antioxidant compounds from different sources^73^. In the present study, a crude ethyl acetate extract of strain BPSAC77 exhibited potent antioxidant activity. The findings agree with earlier reports by Kawamura *et al*.^73^. Kekuda *et al*.^70^ reported that scavenging activity of *Streptomyces* was also increased with increasing concentrations of extract. Moreover, A compound 5-(2, 4- dimethylbenzyl) pyrrolidin-2-one extracted from marine *Streptomyces* sp. VITTK3 exhibited potent antioxidant activity^74^. Similarly, a novel *Streptomyces* Eri12 isolated from the rhizosphere of *Curcumae Longae* demonstrated efficient DPPH radical scavenging activity^75^. Tanvir *et al*.^23^ reported that 66.6% of actinobacteria obtained from traditional medicinal plants showed potent antioxidant activity.

In the present study we detected and quantified several antibiotics including erythromycin, ketoconazole, fluconazole, chloramphenicol, rifampicin and miconazole from endophytic *Streptomyces olivaceu*s strain BPSAC77 and *Streptomyces thermocarboxydus* strain BPSAC147 which showed significant antimicrobial activity. Two other selected strains: *Streptomyces* sp. strain BPSAC101 and *Streptomyces* sp. strain BPSAC121 showed the presence of ketoconazol with a concentration of 289 and 276 μg/g respectively. Among the detected antibiotics fluconazole, chloramphenicol and rifampicin were found for the first time from endophytic *Streptomyces olivaceu*s strain BPSAC77 and *Streptomyces thermocarboxydus* strain BPSAC147. However, in our earlier study we have shown the presence of these three antibiotics in *Streptomyces* sp. strain DBT 204 which is similar with the present findings Passari *et al*.^76^.

Phenolic compounds like catechin, chlorogenic acid, asiatic acid, arjunic acid, gallic acid and boswellic acid were detected and quantified for the first time from the endophytic actinobacterial strain BPSAC77, BPSAC101, BPSAC121 and BPSAC147 which also showed exploitable antimicrobial activity. The greatest yield of gallic acid, a phenolic compound was found in *Streptomyces olivaceus* strain BPSAC77 (92.0 µg/g). This finding was in agreement with like results reported by Gopikrishnan *et al*.^77^ who showed the presence of Gallic acid in *Streptomyces* sp. EC22, obtained from lignocellulose degradation sources. Another phenolic compound chebulagic acid, which is well known to inhibit the growth of *S*. *aureus* and *C*. *albicans*^78^ was reported for the first time from *Streptomyces olivaceus* strain BPSAC77, *Streptomyces* sp. strain BPSAC121 and *Streptomyces thermocarboxydus* strain BPSAC147, and may be responsible for its antimicrobial activities. Similarly, *Streptomyces olivaceus* strain BPSAC77 and *Streptomyces* sp. strain BPSAC101, also showed the presence of kaempferol, a phenolic compound which is used to cure various types of cancers^79,80^. These findings are in accordance with those of Taechowisan *et al*.^26^ who reported kaempferol was detected from endophytic *Streptomyces* sp. strain Tc052 obtained from root tissue of *Alpinia galanga* and exhibited potential antimicrobial activity against *S*. *aureus* ATCC-25932, *E*. *coli* ATCC-10536, *P*. *aeruginosa* ATCC-27853, *B*. *subtilis* ATCC-6633 and *C*. *albicans* ATCC-90028 respectively. Ferulic acid, an anticancer compound was reported only in the isolate *Streptomyces* sp. strain BPSAC121 (2.8 µg/g). This finding was similarly reported by Huang *et al*.^81^ who demonstrated that ferulic acid rich destarched wheat bran hydrolysate resulting from hydrolysis with feruloyl esterases isolated from thermophilic actinomycetes species *Thermobifida fusca* had good antioxidant properties. Paclitaxel (Taxol) is a chemotherapy medicine that was reported for the first time from BPSAC77, BPSAC101 and BPSAC121 strains and is used for the treatment of various types of cancer. Similarly, this active compound was isolated from the actinobacterial strain *Kitasatospora* sp. associated with the *Taxus baccata* plant^15,82^.

## Materials and Methods

### Sample collection

Five disease free ethnomedicinal *R*. *ellipticum* plants were collected from each site; Dampa Tiger Reserve Forest [Dampa TRF] (23°25′N; 92°20′E), and Murlen National Park [MNP] (23°36′N; 93°16′E) Mizoram, India. Plants selected were located within half kilometer range. The roots were harvested at the depth of about 2 feet. To ensure the endophytic nature of isolates, open areas like stems and roots were sealed with wax from both sides. All plant tissues were brought to the laboratory in sterile polythene bags and processed immediately for the isolation and screening of endophytic actinobacteria. Totally, 1050 tissues (350 tissues each from root, stem and leaf) were used to recover endophytic actinobacteria.

### Tissue surface sterilization and isolation of endophytic actinobacteria

Collected plant parts were constantly washed with running tap water to remove soil, organic matter and epiphytic microorganisms. Surface sterilization and validation was carried out according to Passari *et al*.^32^. Briefly, the tissues were dissected into small pieces of 1 cm^2^, rinsed in 0.1% Tween 20 for 10 seconds, and transferred to sterilized petriplates. Tissues were sequentially immersed in 70% ethanol for 2–3 min followed by 0.4% NaOCl for 1 min and 70% ethanol for 2 min. Finally, the tissues were washed three times with double distilled water. Upon sterilization tissues were allowed to dry under laminar air flow and were placed on petri dishes containing nutritional media containing nystatin and cycloheximide (60 μg/ml) to hinder the growth of fungi, and nalidixic acid (60 μg/ml) to avoid the growth of eubacteria were used. The petriplates were incubated at 28 ± 2 °C for 3–4 weeks and microbial growth was recorded and documented. In total, seven nutritional media were used to recover maximum number of actinobacterial isolates: 1: Starch Casein Nitrate Agar (SCNA); 2: Actinomycetes Isolation Agar (AIA); 3: Tap Water Yeast Extract Agar (TWYE); 4: Yeast Malt Extract Agar (ISP2); 5: Glycerol Asparagine Agar (ISP5); 6: Tyrosine agar (ISP7) and 7: Streptomyces agar (SA).

### Preliminary identification of Actinobacteria

Cultural and morphological characteristics were determined after growth at 28 °C for 2–4 weeks as per International Streptomyces Project^83^. The isolates were identified upto genus level by observing morphology and color of aerial and substrate mycelium, characteristics of colonies on the plate, spore mass color, color of diffusible pigments and spore chain morphology^84^. Colony morphological features were observed using light microscopy to note their hyphal length and structure, to assign them as separate isolates. The acid production was determined using basal inorganic nitrogen medium containing 1 g of (NH_4_)_2_HPO_4_; 0.02 g of KCl; 0.2 g of MgSO_4_ 0.7H_2_O per litre of dH_2_O. The pH of the medium was adjusted to 7.0 before the addition of 15 ml of a 0.04% solution of bromocresol purple. After autoclaving, 0.5 ml of a10% solution of each carbohydrate was added to the medium. Then, few drops of endophytic actinobacterial broth suspension was added to the tube and incubated at 28 °C for 2–3 weeks. The formation of a yellow color was considered as positive for acid production. The following carbohydrates were used: fructose, galactose, inositol, lactose, mannitol, rhamnose, trehalose, and xylose^85^.

### Determination of antimicrobial potential

In total eight pathogens were selected: Pseudomonas aeruginosa (MTCC-2453), Staphylococcus aureus (MTCC-96), Escherichia coli (MTCC-739), Bacillus subtilis (NCIM-2124), Candida albicans (MTCC-3017), Fusarium oxysporum f. sp. ciceri (MTCC-2791), Fusarium proliferatum (MTCC-286) and Fusarium oxysporum (MTCC-284) were used to determine antimicrobial potential. All the tested pathogens were obtained from Microbial Type Culture Collection (IMTECH), India and were maintained in nutrient agar and potato dextrose agar media as specified.

The obtained isolates were screened for their antibacterial potential using agar well diffusion method as per Saadoun and Muhana^86^. Briefly, the isolates were grown in ISP1 media and incubated at 28 °C, 250 rpm for 7–10 days. The bacterial cells were harvested by centrifugation at 10,000 rpm for 5 min and the supernatant was collected for testing the antibacterial activity. The bacterial pathogen was spread on modified nutrient agar plate and a 6 mm diameter of wells was removed by using sterile cork borer. In every well, 50 µl of endophytic actinobacterial supernatant was added and incubated at 37 °C for 24 h. The suppression of pathogenic bacterial growth was measured seeing the zone of inhibition.

All isolates were screened for their antagonistic activity using a dual culture method as per Bredholdt *et al*.^87^. Briefly, a 5 mm potato dextrose agar (PDA) block with fungal culture was placed at the center of a petri plate containing PDA and the actinobacterial isolates were streaked at the periphery of the plates. The plates were incubated at 28 °C for 7–14 days and the diameter of fungal mycellial growth was measured and compared to the control (without any actinobacterial isolate). Plates with fungal pathogen alone served as the control and the percentage of inhibition was calculated: % inhibition = [1-(Fungal growth/Control growth)] × 100.

### Amplification of 16S rRNA gene and sequencing

Based on antimicrobial ability, 81 isolates were identified using 16S rRNA gene amplification and sequencing. Extraction of genomic DNA was performed using the In-vitrogen pure link DNA isolation kit (Catalogue No: K182002). 16S rRNA gene amplification was carried out as per Cui *et al*.^88^. PCR reactions were carried out in a total volume of 25 µl consisting of 0.5 µl of template (50 ng), 0.4 µl of each primer (10 pmol), 2.0 µl of dNTPs (2.5 mM each), 2.5 µl of 1x PCR buffer, 0.2 µl of Taq DNA polymerase (5 U/µl). PCR conditions were: initial denaturation at 95 °C for 4 min, followed by 30 cycles of denaturation at 94 °C for 1 min, annealing at 57 °C for 1 min and extension at 72 °C for 1.2 min with a final extension step at 72 °C for 10 min. The PCR products were analyzed using 1.2% of agarose gel. The gels were visualize under UV light and documented using a Bio-rad Gel Doc XR + system (Hercules, CA, USA). The obtained PCR products were purified using the In-vitrogen Pure Link kit (Catalogue No: K310001), and were sequenced commercially at Sci-Genome Pvt. Ltd. Kochin, India.

### Phylogenetic analysis

The 16S rRNA gene sequences obtained was compared with existing sequences in the NCBI database using the BLASTn search program. The sequence alignment was performed using Clustal W in the MEGA 5.05 software package^89^. Suitable models with lowest BIC scores (Bayesian Information Criterion) and highest AICc values (Akaike Information Criterion, corrected) were selected using MEGA 5.0^90^.The phylogenetic tree was constructed by maximum likelihood method using MEGA 5.05 with Kimura 2-parameter model (K2 + I). The reliability of the phylogenetic tree was tested by bootstrap analysis using 1,000 replicates^91^.

### Screening for plant growth promoting traits

#### Phosphate solubilization

Screening for phosphate solubilization efficiency of endophytic actinobacterial isolates was carried out by streaking the isolates on PKV agar media as described by Bashan *et al*.^49^. The streaked plates were incubated at 28 °C for 7–15 days and observed for a clearing zone around the colonies. The phosphate solubilization efficiency (PSE) was calculated based on the clear zones formed around the colonies.

#### Indole acetic acid (IAA) and ammonia production

The IAA production potential was calculated as per Gordon and Weber^92^. The endophytic actinobacterial isolates were grown on ISP2 broth containing 0.2% L-tryptophan incubated at 28 °C with shaking at150 rpm for 7–14 days and was centrifuged at 11,000 rpm for 15 min. Development of a pink-red color confirms IAA production by the addition of 0.5% of Salkowski reagent into 1 ml of cell free supernatant. Estimation of IAA was measured by taking the absorbance at 530 nm using a spectrophotometer (Thermo scientific, Multiskan GO) and the amount of IAA was calculated in µg/ml as compared with the standard curve of IAA.

Ammonia production was estimated as demonstrated by Cappucino and Sherman^93^. The endophytic actinobacterial isolates were grown in peptone water broth incubated at 28 °C with shaking at 150 rpm for 7–14 days. The grown broth was added with 0.5 ml of Nesseler’s reagent and development of a brown to yellow color confirms ammonia production. The absorbance was taken at 530 nm using a spectrophotometer (Thermo scientific, Multiskan GO) and ammonia production was expressed in mg/ml when compared with the standard curve of (NH_4_)_2_SO_4_.

#### Production of hydrolytic enzymes

Cellulolytic activity of endophytic actinobacteria was determined by streaking the isolates on CMCase agar medium containing (gL-1) [K2HPO4–0.5 g, MgSO4.7H2O-0.25 g, carboxymethyl cellulose-15 g and agar-20 g). The isolates were single streaked in CMC agar medium and incubated at 28 °C for 7–14 days. After growing, the plates were flooded with an aqueous solution of Congo red solution (1 mg/ml) for 15 min and distained with 1 M NaCl for 15 min respectively. A clear zone of hydrolysis showed the production of CMCase^94^.

A amylase activity was determined using soluble starch as a substrate using GYP media containing (gL-1) [glucose- 5.0 g; peptone- 5.0 g; yeast extract- 3.0 g; soluble starch-1.0 g and agar −20.0 g]. The endophytic actinobacterial isolates were single streaked in GYP agar medium and incubated at 28 °C for 7–14 days. Further, the plates were flooded with 5 ml of 1% iodine solution for 15 min and a clear zone around the colonies indicated amylase production^95^.

#### Quantitative estimation of CMCase and amylase

The positive isolates that showed the production of CMCase and amylase were selected for quantitative estimation of the enzymes. Cellulase and amylase activities were evaluated using the DNS method as described earlier Passari *et al*.^43^. The endophytic isolates were centrifuged at 5,000 rpm for 10 min, and the cell free supernatant was collected to carry out enzymatic assay. Briefly, 0.5 ml of cell free supernatant was mixed with 0.5 ml of specific substrates of carboxymethyl cellulose for cellulase activity and, soluble starch for amylase activity in 50 mM of phosphate buffer (pH-6.9). The mixture was incubated at 50 °C for 30 min and the reaction was stopped by the addition of 3 ml of 3, 5-dinitrosalicylic acid (DNS) reagent. The mixture was boiled for 10 min and then 1 ml of sodium potassium tartarate (KNaC_4_H_4_O_6_·4H_2_O) was added. The absorbance was recorded at 540 nm using a spectrophotometer (Thermo scientific Multiskan GO microplate reader). The enzyme activities were estimated using a standard graph of glucose.

#### Antibiotic resistance profiling

The antibiotic resistance pattern of all the isolates were estimated against 12 antibiotics: vancomycin (30 µg/ml), gentamicin (10 μg/ml), penicillin G (10 μg/ml), norfloxcin (30 μg/ml), tetracycline (30 μg/ml), nalidixic acid (30 μg/ml), ampicillin (10 μg/ml), chloramphenicol (30 μg/ml), erythromycin (15 μg/ml), streptomycin (30 μg/ml), rifampicin (30 μg/ml) and nystatin (30 μg/ml). The choice of antibiotics includes both narrow and broad spectrum antibiotics, and the concentrations used are the highest inhibitory concentrations as per Williams *et al*.^96^. Isolates were grown on Muller-Hinton agar media and growth was checked for 72 h, and considered as sensitive (S), intermediate (I) or resistant (R) to an antibiotic.

#### Detection of biosynthetic genes

Polyketide synthase genes (PKS I and PKS II) were amplified using degenerate primers: (K1F: 5′-TSAAGTCSAACATCGGBCA-3′ and M6R: 5′-CGCAGGTTSCSGTACCAGTA-3′ and KS∞: 5′-TSGCSTGCTTGGAYGCSATC-3′ and KSβ: 5′-TGGAANCCGCCGAABCCTCT-3′)^97^ and Non ribosomal peptide synthetase (NRPS) adenylation domain was amplified with a set of degenerate primers (A3F: 5′-GCSTACSYSAT STACACSTCSGG-3′ and A7R: 5′-SASGTCVCCSGTSGCGTAS- 3′)^98^.

### Expression of antimicrobial biosynthetic genes (PKSI and NRPS) using *Real Time-qPCR*

#### RNA isolation and two step *Real Time-qPCR*

Total RNA was isolated from cells of actively growing potential strains: *Streptomyces olivaceus* strain BPSAC77, *Streptomyces* sp. strain BPSAC121 and *Streptomyces thermocarboxydus* strain BPSAC147, at different time intervals using a NucleoSpin RNA isolation kit (MACHEREY-NAGAL, Germany). One microgram of RNA was reverse-transcribed to cDNA with SensiFAST^TM^cDNA synthesis kit (Bioline, UK) following the manufacturers protocol. Quantitative amplification of cDNAs from reference genes and target genes were done by a StepOne-PlusTM Real Time PCR System (Applied Biosystems, USA) using SYBR Green Real-Time PCR master mix (Thermo Fisher Scientific, USA). cDNAs were replaced by sterile water for a template control reaction. The PKS-I and NRPS of biosynthetic coding clusters was taken as a target and the 16S rRNA gene was used an endogenous control throughout the experiment. The PCR amplification was performed using following reaction conditions: initial denaturation of 95 °C for 10 min (1 cycle), followed by 40 cycles of 95 °C for 15 s, 60 °C for 1 min and post PCR, melt curve analysis of 95 °C for 1 min followed by 55 °C for 30 s and a final ramp to 95 °C with continuous data collection (1cycle) to test for primer dimer and nonspecific amplification.

#### Preparation of ethyl acetate extract of potent actinobacterial isolates

Isolates that showed significant plant growth promoting abilities were inoculated in ISP2 broth (yeast malt extract broth) and maintained at 28 °C, 140 rpm for 14 days. The fully-grown culture was filtered using Whatman filter paper No. 1 and the cell free culture filtrate was extracted trice with ethyl acetate followed by evaporation to dryness under pressure at 45 °C (Rotary evaporator, BUCHI, India).

#### Determination of antioxidant activity by DPPH assay

Antioxidant potential was determined using a 2, 2-diphenyl-1-picrylhydrazyl radical (DPPH) assay per Savran *et al*.^99^; Zengin *et al*.^100^. Briefly, 50 µl test sample and 150 µl of 50 mM freshly prepared DPPH solution were added in a 96-well micro-plate and incubated at 28 °C in the dark for 30 min. Reduction of DPPH radicals was measured at 517 nm using a spectrophotometer (Thermo scientific, Multiskan GO) with methanol as the blank. Ascorbic acid was used as a positive control and the activities were expressed by IC_50_, which was represented as the dilution of crude extract to produce 50% inhibition of DPPH and estimated by linear regression. All samples were assayed in triplicate. The percentage scavenging of DPPH by the extracts was calculated as:

$$ \% \,{\rm{DPPH}}\,{\rm{Radical}}\,{\rm{scavenging}}=[({\rm{Ac}}-{\rm{At}})/{\rm{Ac}}]\times 100$$

where, Ac = absorbance of the control (DPPH); At is the absorbance of test sample.

### Detection and quantification of bioactive compounds using UHPLC-QqQ_LIT_-MS/MS

#### Materials and Preparation of standard solution

Acetonitrile, methanol (HPLC grade), standard bioactive compounds and formic acid (analytical grade) were purchased from Fluka, Sigma-Aldrich (St. Louis, MO, USA). Ultra-pure water was obtained from a Direct-Q 8 UV water purification system (EMD Millipore Corporation, Billerica, MA, USA). A mixed standard stock solution (1 mg/mL) of sixteen bioactive compounds which includes six antibiotics (erythromycin, ketoconazole, fluconazole, chloramphenicol, rifampicin and miconazole), nine phenolic compounds (catechin, kaempferol, chebulagic acid, chlorogenic acid, Asiatic acid, ferulic acid, arjunic acid, gallic acid and boswellic acid) and an anticancerous compound, Paclitaxel were prepared in methanol. The working standard solutions were then prepared by appropriate dilution of the mixed standard solution with acetonitrile to a series of concentration ranges from 0.1–1000 ng/mL. The standard stock and working solutions were stored at −20 °C until use and vortexed for 30 s prior to injection.

#### UHPLC-QqQ_LIT_-MS/MS Conditions

Compounds were separated using an AcquityBEH C18 (2.1 mm × 50 mm, 1.7 µm; Waters, Milford, MA) column and quantified as per Passari *et al*.^76^. Quantitative analysis was performed using multiple reactions monitoring (MRM) acquisition mode at the unit resolution. The optimized ion source parameters for negative mode was as follows: ion spray voltage, −4200 V; curtain (CUR) gas, 20 psi; nebulizer gas (GS1) and heater gas (GS2), 20 psi; ion source temperature, 350 °C; collision activated dissociation (CAD) gas, medium and the interface heater was on. The optimized compound dependent MRM parameters [declustering potential (DP), entrance potential (EP), collision energy (CE) and cell exit potential (CXP)] for precursor-to-product ion transition of each analyte are presented in Table 2.

**Table 2:** Multiple reaction monitoring (MRM) compound dependent parameters for reference analytes among four selected strains.

Sl. No.	Analytes	*rt* (min)	Q1 Mass (Da)	Q3 Mass (Da)	DP (V)	EP (V)	CE (eV)	CXP (V)	Polarity
1	Fluconazole	1.31	307.1	220.1	59	10	27	8	Positive
2	Chloramphenicol	1.61	322.1	153.1	−56	−7	−24	−27	Negative
3	Erythromycin	1.57	735.8	577.4	80	6	25	13	Positive
4	Ketoconazole	1.82	532.1	82.0	106	10	68	10	Positive
5	Rifampicin	2.24	823.5	791.4	53	9	24	19	Positive
6	Miconazole	2.34	417.1	159.1	168	10	42	24	Positive
7	Catechin	1.50	289.0	203.0	−110	−10	−29	−8	Negative
8	Kaempferol	1.80	285.0	239.0	−95	−5	−39	−15	Negative
9	Chabulagic acid	2.40	431.0	311.0	−91	−6	−26	−7	Negative
10	Chlorogenic acid	2.50	353.5	301.1	−176	−10	−10	−19	Negative
11	Asiatic acid	2.55	487.1	409.6	−145	−10	−60	−28	Negative
12	Ferulic acid	2.70	193.0	134.0	−58	−5	−23	−9	Negative
13	Arjunic acid	3.50	487.3	423.3	−117	−6	−47	−20	Negative
14	Gallic acid	3.62	169.0	125.0	−59	−8	−21	−10	Negative
15	Paclitaxel	3.76	852.3	525.1	−57	−9	−17	−16	Negative
16	Boswellic acid	4.24	455.5	377.0	−19	−8	−48	−22	Negative

[Precursor ion (Q1), product ion (Q3), declustering potential (DP), entrance potential (EP), collision energy (CE) and cell exit potential (CXP)].

#### Statistical analysis

The data (mean ± SD) was calculated using Microsoft Excel XP 2008, and one-way ANOVA was performed using SPSS software 20.0. Threshold cycle (C_T_) was compared with log_10_ relative copy number of the sample from a dilution series. The expression level calculated by the formula 2^−ΔΔCt^ represents the x-fold difference from the calibrator.

### Data availability statement

All data generated or analysed during this study are included in this published article (and its Supplementary Information files).

## Electronic supplementary material

Supplementary Information

## References

[1] El-Shatoury SA (2013). Generic and functional diversity in endophytic actinomycetes from wild Compositae plant species at South Sinai—Egypt. Res. Microbiol..

[2] Gaiero JR (2013). inside the root microbiome: bacterialroot endophytes and plant growth promotion. Am. J. Bot..

[3] Rai, M., Agarkar, G. & Rathod, D. Multiple applications of endophytic *Colletotrichum* species occurring in medicinal plants, in novel plant bioresources: Applications in food, medicine and cosmetics. In: Gurib-Fakim. A. (ed.) Novel plant bioresources. Wiley, Chichester, pp 227–236 (2014a).

[4] Rai M (2014). Fungal growth promotor endophytes: apragmatic approach towards sustainable food and agriculture. Symbiosis.

[5] Donn S, Kirkegaard JA, Perera G, Richardson AE, Watt M (2014). Evolution of bacterial communities in the wheat crop rhizosphere. Environ. Microbiol..

[6] Bulgarelli D (2012). Revealing structure and assembly cues for Arabidopsis root-inhabiting bacterial microbiota. Nature.

[7] Lundberg DS (2012). Defining the core *Arabidopsis thaliana* root microbiome. Nature.

[8] Peiffer JA (2013). Diversity and heritability of the maize rhizosphere microbiome under field conditions. PNAS.

[9] Shakya M (2013). Comparative metagenomic and rRNA microbial diversity characterization using archaeal and bacterial synthetic communities. Environ. Microbiol..

[10] Bonito GM (2014). Plant host and soil origin influence fungal and bacterial assemblages in the roots of woody plants. Mol. Ecol..

[11] Edwards J (2015). Structure, variation, and assembly of the root-associated microbiomes of rice. PNAS.

[12] Agler MT (2016). Microbial Hub Taxa Link Host and Abiotic Factors to Plant Microbiome Variation. PloS Biol..

[13] Coleman-Derr D (2016). Plant compartment and biogeography affect microbiome composition in cultivated and native *Agave* species. New Phytol..

[14] Wagner MR (2016). Host genotype and age shape the leaf and root microbiomes of a wild perennial plant. Nat. Commun..

[15] Golinska P (2015). Endophytes actinobacteria of medicinal plants: diversity and bioactivity. Anton. Van. Leeuwen..

[16] Nimnoi P, Pongsilp N, Lumyong S (2010). Endophytic actinobacteria isolated from *Aquilaria crassna* Pierre ex Lec and screening of plant growth promoter’s production. World J. Microbiol.Biotechnol..

[17] Qin S (2012). Abundant and diverse endophytic actinobacteria associated with medicinal plant *Maytenus austroyunnanensis* in Xishuangbanna tropical rainforest revealed by culture-dependent and culture-independent methods. Environ. Microbiol. Rep..

[18] Zhao K, Penttinen P, Xiao TGJ, Chen Q, Xu J (2011). The Diversity and antimicrobial activity of endophytic actinobacteria isolated from medicinal plants in Panxi Plateau,China. Curr. Microbiol..

[19] Verma VC (2009). Endophytic Actinobacteria from *Azadirachta indica A*. Juss.: Isolation, Diversity, and Anti-microbial Activity. Microb. Ecol..

[20] Gohain A (2015). Antimicrobial biosynthetic potential and genetic diversity of endophytic actinomycetes associatedwith medicinal plants. FEMS Microbiol. Lett..

[21] Zhang X, Ren K, Zhang L (2012). Screening and preliminary identification of medicinal plants endophytic actinomycetes used for inhibiting penicillin-resistant *Staphylococcus aureus*. Int. J. Biol..

[22] Wang P (2014). Alkaloids from the mangrove-derived actinomycete *Jishengella endophytica* 161111. Mar. Drug..

[23] Tanvir R, Sajid I, Hasnain S (2014). Larvicidal potential of Asteraceae family endophytic actinomycetes against *Culex quinquefasciatus* mosquito larvae. Nat. Prod. Res..

[24] Castillo U (2002). Munumbicins, wide- spectrum antibiotics producedby *Streptomyces* NRRL 30562, endophytic on *Kennedia nigriscans*. Microbiol..

[25] Castillo UF (2006). Munumbicins E-4 and E-5: novel broad-spectrum antibiotics from *Streptomyces* NRRL 3052. FEMS Microbiol. Lett..

[26] Taechowisan T, Chuaychot N, Chanaphat S, Wanbanjob A, Shen Y (2008). Biological activity of chemical constituents isolated from *Streptomyces* sp. Tc052, an endophyte in *Alpinia galanga*. Int. J. Pharmacol..

[27] Akshatha VJ, Nalini MS, D’Souza C, Prakash HS (2014). *Streptomycete* endophytes from anti-diabetic medicinalplants of the Western Ghats inhibit alpha-amylase andpromote glucose uptake. Lett. Appl. Microbiol..

[28] Zhang J (2014). A new prenylated indole derivative from endophytic actinobacteria *Streptomyces* sp. neau-D50. Nat. Prod. Res..

[29] Doroghazi JR, Metcalf WW (2013). Comparative genomics of actinomycetes with a focus on natural product biosynthetic genes. BMC Genomic..

[30] Goodfellow M, Fiedler HP (2010). A guide to successful bioprospecting:informed by actinobacterial systematics. Antvan. Leeuw..

[31] Janso JE, Carter GT (2010). Biosynthetic potential of phylogenetically unique endophytic actinomycetes from tropical plants. Appl. Environ. Microbiol..

[32] Passari AK, Mishra VK, Saikia R, Gupta VK, Singh BP (2015). Isolation, abundance and phylogenetic affiliation of endophytic actinobacteria associated with medicinal plants and screening for their *in vitro* antimicrobial biosynthetic potential. Front. Microbiol..

[33] McMillan M, Pereg L (2014). Evaluation of Reference Genes for Gene Expression Analysis Using Quantitative RT-PCR in *Azospirillum brasilense*. PLoSONE.

[34] Gao W, Zhang W, Meldrum DR (2013). RT-qPCR based quantitative analysis of gene expression in single bacterial cells. J. Microbiol. Methods.

[35] Sharma HK, Chhangte L, Dolui AK (2001). Traditional medicinal plants in Mizoram, India. Fitoterapia..

[36] Qin S, Xing K, Jiang JH, Xu LH, Li WJ (2011). Biodiversity, bioactive natural products and biotechnological potential of plant associated endophytic actinobacteria. Appl. Microbiol Biotechnol..

[37] Castillo U (2003). Kakadumycins, novel antibiotics from *Streptomyces* sp. NRRL 30566, an endophyte of *Grevillea pteridifolia*. FEMS Microbiol. Lett..

[38] Cao L (2004). Isolation of Endophytic Actinomycetes from roots and leaves of Banana (*Musa acuminate*) Planta and their activities against *Fusarium oxysporum* f.sp. *cubense*. World J. Microbiol. Biotech..

[39] Qin S, Li J, Chen HH, Zhao GZ, Zhu WY (2009). Isolation, Diversity and antimicrobial activity of rare actinobacteria from medicinal plants of tropical rain forests in Xishuangbanna, China. Appl. Environ. Microbiol..

[40] Taechowisan T, Peberdy JF, Lumyong S (2003). Isolation of endophytic actinobacteria from selected plants and their antifungal activity. World J. Microbiol. Biotechnol..

[41] Cao L, Qiu Z, You J, Tan H, Zhou S (2005). Isolation and characterization of endophytic *Streptomycete* antagonistics of *Fusarium* wilt pathogen from surface-sterilized banana roots. FEMS Microbiol. Lett..

[42] Wu CP (2006). Studies on the chemical components and antibacterial activity of volatile oil of *Mosla dianthera maxim*. J. Fujian. Norm. Univ (Nat. Sci. Ed.).

[43] Passari AK (2016). Phytohormone production endowed with antagonistic potential and plant growth promoting abilities of culturable endophytic bacteria isolated from *Clerodendrum colebrookianum* Walp. Microbiol. Res..

[44] Mingma R, Pathom-aree W, Trakulnaleamsai S, Thamchaipenet A, Duangmal K (2014). Isolation of rhizospheric and roots endophytic actinobacteria from Leguminosae plant and their activities to inhibit soybean pathogen, *Xanthomonas campestris*pv. Glycine. World J. Microbiol. Biotechnol..

[45] Baltz RH (2006). Is our antibiotic pipeline unproductive because of starvation, constipation or lack of inspiration?. J. Ind. Microbiol. Biotechnol..

[46] Graner G, Persson P, Meijer J, Alstrom S (2003). A study on microbial diversity in different cultivars of *Brassica napus* in relation to its wilt pathogen, *Verticillium longisporum*. FEMS Microbiol. Lett..

[47] Rosenberg, E. The Prokaryotes: Actinobacteria. In: Delong, E. F., Lor, S., Stackebrandt, E. & Thompson, F. (ed.). Springer-Verlag Berlin, Heidelberg, ISBN 978-3-642-30139-1, 4^th^ edition (2014).

[48] Pradhan N, Sukla LB (2006). Solubilization of inorganic phosphates by fungi isolated from agriculture soil. Afr. J. Biotechnol..

[49] Bashan Y, Kamnev AA, De-Bashan LE (2013). Tricalcium phosphate is inappropriate as a universal selection factor for isolating and testing phosphate solubilizing bacteria that enhance plant growth: a proposal for an alternative procedure. Biol. Ferti. Soils.

[50] Mehta S, Nautiyal SC (2001). An efficient method for qualitative screening of phosphate-solubilizing bacteria. Curr. Microbiol..

[51] Hamdali H, Hafidi M, Virolle MJ, Ouhdouch Y (2008). Growth promotion and protection against damping-off of wheat by two rock phosphate solubilizing actinobacteria in a P-deficient soil under greenhouse conditions. Appl. Soil Ecol..

[52] Passari AK (2015). *In Vitro* and *In Vivo* Plant Growth Promoting Activities and DNA Fingerprinting of Antagonistic Endophytic Actinobacteria associates with Medicinal Plants. PloS ONE..

[53] Ribeiro CM, Cardoso EJ (2102). Isolation, selection and characterization of root-associated growth promoting bacteria in Brazil Pine (*Araucaria angustifolia*). Microbiol. Res..

[54] Khamna S, Yokota A, Lumyong S (2009). Actinobacteria isolated from medicinal plant rhizosphere soils: diversity and screening of antifungal compounds, indole-3-acetic acid and siderophore production. World J. Microbiol. Biotechnol..

[55] Zahir A, Abbas SA, Khalid M, Arshad M (2000). Structure dependent microbially derived plant hormones by improving growth of maize seedlings. Pak. J. Biol. Sci..

[56] Patten C, Glick BR (1996). Bacterial biosynthesis of indole-3-acetic acid. Can. J. Microbiol..

[57] Minaxi LN, Yadav RC, Saxena J (2012). Characterization of multifaceted *Bacillus* sp. RM-2 for its use as plant growth promoting bioinoculant for crops grown in semi arid deserts. Appl. Soil Ecol..

[58] Marques APGC, Pires C, Moreira H, Rangel AOSS, Castro PML (2010). Assessment of the plant growth promotion abilities of six bacterial isolates using *Zea mays* as indicator plant. Soil Biol. Biochem..

[59] Kavamura VN (2013). Screening of Brazilian cacti rhizobacteria for plant growth promotion under drought. Microbiol. Res..

[60] Xing-hua L (2009). The most stirring technology in future: cellulase enzyme and biomass utilization. J. Microbiol. Biotechnol..

[61] Sirisha B, Haritha R, Mohan YSYVJ, Kumar KS, Ramana T (2013). Bioactive compounds from marine actinobacteria isolated from the sediments of Bay of Bengal. Int. J. Phar. Chem. Biolog. Sci..

[62] Jang HD, Chen KS (2003). Production and characterization of thermostable cellulases from *Streptomyces* transformant T3-1. World J. Microbiol. Biotechnol..

[63] Azad MA (2009). Isolation and characterization of a novel thermostable alpha-amylase from Korean pine seeds. N. Biotechnol..

[64] Rajagopalan G, Krishnan C (2008). Alpha-amylase production from catabolite derepressed *Bacillus subtilis* KCC103 utilizing sugarcane bagasse hydrolysate. Bioresour. Technol..

[65] Pal A, Chattopadhyay A, Paul AK (2012). Diversity and antimicrobial spectrum of endophytic bacteria isolated from *Paederia Foetida* L. Int. J. Curr. Pharm. Res..

[66] Falcao JP (2004). Virulence characteristics and epidemiology of *Yersinia enterocolitica* and Yersiniae other than *Y*. *pseudotuberculosis* and *Y*. *pestis* isolated from water and sewage. J. Appl. Microbiol..

[67] Rebouças RH (2011). Antimicrobial resistance profile of *Vibrio* species isolated from marine shrimp farming environments (*Litopenaeus vannamei*) at Ceara, Brazil. Environ. Res..

[68] Awah FM (2010). Free radical scavenging activity and immunomoduatory effect of *Stachytarpheta angustifolia* leaf extract. Food Chem..

[69] Chung Y, Chien C, Teng K, Chou S (2006). Antioxidative and mutagenic properties of *Zanthoxylum ailanthoides* Siebandzucc. Food Chem..

[70] Kekuda PTR, Shobha KS, Onkarappa R (2010). Studies on antioxidant and antihelmintic activity of two *Streptomyces* species isolated from Western Ghat soil of Agumbe, Karnataka. J. Pharm. Res..

[71] Reuter S, Gupta SC, Chaturvedi MM, Aggarwal BB (2010). Oxidative stree, inflammation and cancer: how are they linked?. Free Radic. Biol. Med..

[72] Jomova K, Valko M (2011). Importance of iron chelation in free radical induced oxidative stress and human disease. Curr. Pharm. Des..

[73] Kawamura T (2012). JBIR-94 and JBIR-125, antioxidative phenolic compound from *Streptomyces* sp. R56-07. J. Nat. Prod..

[74] Thenmozhi M, Sindhura S, Kannabiran K (2010). Characterization of antioxidant activity of *Streptomyces* species VITTK3 isolated from Puducherry Coast, India. J. Adv. Sci. Res..

[75] Zhong K (2011). Antioxidant activity of novel *Streptomyces* strain Eri12 isolated from the rhizosphere of *Rhizoma curcuma* Longae. Curr. Res. Bacteriol..

[76] Passari AK (2016). Detection of biosynthetic gene and phytohormone production by endophytic actinobacteria associated with *Solanum lycopersicum* and their plant growth- promoting effect. Res. Microbiol..

[77] Gopikrishnan V, Radhakrishnan M, Shanmugasundaram M, Pazhanimurugan R, Balagurunathan R (2016). Antibiofouling potential of quercetin compound from marine-derived actinobacterium, *Streptomyces fradiae* PE7 and its characterization. Environ. Sci. Pollut. Res..

[78] Shin-ichi H (1997). Immunosuppressive Effects of Gallic Acid and Chebulagic Acid on CTL-Mediated Cytotoxicity. Biol. Pharm. Bull..

[79] Calderon-Montaño JM, Burgos-Moron E, Perez-Guerrero C, Lopez-Lazaro M (2011). A review on the dietary flavonoid kaempferol. Mini. Rev. Med. Chem..

[80] Kim SH, Choi KC (2013). Anti-cancer Effect and Underlying Mechanism(s) of Kaempferol, a Phytoestrogen, on the Regulation of Apoptosis in Diverse Cancer Cell Models. Toxicol. Res..

[81] Huang YC (2013). Production and antioxidant properties of the ferulic acid rich destarched wheat bran hydrolysate by feruloyl esterases from thermophilic actinomycetes. BioResources..

[82] Caruso M (2000). Isolation of endophytic fungi and actinomycetes taxane producers. Ann. Microbiol..

[83] Shirling EB, Gottlieb D (1966). Methods for characterization of *Streptomyces* species. Int. J. Syst. Bacteriol..

[84] Goodfellow, M. & Haynes, J. A. Actinomycetes in marine sediments. In: Ortiz-Ortiz, L., Bojalil, L. F. & Yakoleff, V. (ed.). *Biological*, *biochemical*, *and biomedical aspects of actinomycetes*. New York, Academic Press p. 453–472 1984).

[85] Gordon RE, Barnett DA, Handerhan JE, Pang CHN (1974). *Nocardia coeliaca*, *Nocardia autotrophica* and the *Nocardin* strains. Int. J. Syst. Bacteriol..

[86] Saadoun I, Muhana A (2008). Optimal production conditions, extraction, partial purification and characterization of inhibitory compound(s) produced by *Streptomyces* Ds-104 isolate against multi-drug resistant *Candida albicans*. Curr. Trends Biotechnol. Pharm..

[87] Bredholdt H (2007). Rare actinomycete bacteria from the shallow water sediments of the Trondheim fjord, Norway: isolation, diversity and biological activity. Environ. Microbiol..

[88] Cui XL (2001). *Streptomonospora* gen. nov., a new member of the family Nocardiopsaceae. Int. J. Syst. Evol. Microbiol..

[89] Tamura K (2011). MEGA5: molecular evolutionary genetics analysis using maximum likelihood, evolutionary distance, and maximum parsimony methods. Mol. Biol. Evol..

[90] Nei, M. & Kumar, S. *Molecular evolution and phylogenetics*. Oxford University Press, New York (2000).

[91] Felsenstein J (1985). Confidence limits of phylogenies: An approach using the bootstrap. Evol..

[92] Gordon SA, Weber RP (1951). Colorimetric estimation of indole acetic acid. Plant Physiol..

[93] Cappucino, J.C. & Sherman, N. *Microbiology: a laboratory manual*. New York, Benjamin: Cummings Publishing Company, 125–179 (1992).

[94] Gupta, P., Samant, K. & Sahu, A. Isolation of Cellulose-Degrading Bacteria and Determination of Their Cellulolytic Potential. *Int*. *J*. *Microbiol*., doi:10.1155/2012/578925 (2012). 10.1155/2012/578925 PMC3270400 22315612

[95] Hankin L, Anagnostakis SL (1975). The use of solid media for detection of enzyme production by fungi. Mycologia..

[96] Williams, S.T., Sharpe, M.E. & Holt, J.G. Bergey’s Manual of Systematic Bacteriology. Vol.4.Baltimore, MD: Williams and Wilkins (1989).

[97] Ayuso-Sacido A, Genilloud O (2005). New PCR primers for the screening of NRPS and PKS-I systems in actinobacteria: detection and distribution of these biosynthetic gene sequences in major taxonomic groups. Microb. Ecol..

[98] Metsa-Ketela M (1999). An efficient approach for screening minimal PKS genes from. Streptomyces. FEMS. Microbiol. Lett..

[99] Savran A (2016). Phenolic compounds and biological effects of edible *Rumex scutatus* and *Pseudosempervivum sempervivum*: potential sources of natural agents with health benefits. Food Funct..

[100] Zengin G (2017). *Euphorbia denticulata* Lam.: A promising source of phyto-pharmaceuticals for the development of novel functional formulations. Biomed. Pharmacother..

