# Author Correction: Insights into the functionality of endophytic actinobacteria with a focus on their biosynthetic potential and secondary metabolites production

**DOI:** 10.1038/s41598-018-22947-w

**Published:** 2018-03-12

**Authors:** Ajit Kumar Passari, Vineet Kumar Mishra, Garima Singh, Pratibha Singh, Brijesh Kumar, Vijai Kumar Gupta, Rupak Kumar Sarma, Ratul Saikia, Anthonia O’. Donovan, Bhim Pratap Singh

**Affiliations:** 1 Molecular Microbiology and Systematics Laboratory, Department of Biotechnology, Mizoram University, Aizawl, Mizoram 796004 India; 2 SAIF, CSIR-Central Drug Research Institute (CSIR-CDRI), Lucknow, 226012 India; 3 Department of Chemistry and Biotechnology, ERA Chair of Green Chemistry, Tallinn University of Technology, 12618 Tallinn, Estonia; 4 Biotechnology Division, CSIR-NEIST, Jorhat, Assam 785006 India; 5 Applied Biology and Biopharmaceutical Science, School of Science & Computing, Galway-Mayo Institute of Technology, Galway, Ireland

Correction to: *Scientific Reports* 10.1038/s41598-017-12235-4, published online 18 September 2017

The original version of this article contained a typographical error in the spelling of the author Rupak Kumar Sarma, which was incorrectly given as Rupak Kumar Sharma.

In addition, this article also contained an error in the Affiliation 4 which was incorrectly listed as ‘Biotechnology Division, CSIR-NEIST, Jorhat, Assam, 892890, India’. The correct affiliation is listed below:

Biotechnology Division, CSIR-NEIST, Jorhat, Assam, 785006, India.

These errors have now been corrected in the PDF and HTML versions of the article and in the supplementary information that now accompanies the article.

