# Supplementary material for: Insights into the functionality of endophytic actinobacteria with a focus on their biosynthetic potential and secondary metabolites production
Source: Sci Rep. 2017 Sep 18;7:11809. doi: 10.1038/s41598-017-12235-4 (PMC5603540; doi:10.1038/s41598-017-12235-4)
Supplement: Supplementary file 1 — Supplementary Information [file 41598_2017_12235_MOESM1_ESM.pdf]

Insights into the functionality of endophytic actinobacteria with a focus on their biosynthetic potential and secondary metabolites production

Ajit Kumar Passari[1], Vineet Kumar Mishra[1], Garima Singh[1], Pratibha Singh[2], Brijesh Kumar[2], Vijai Kumar Gupta[3], Rupak Kumar Sarma[4], Ratul Saikia[4], Anthonia O' Donovan[5] and Bhim Pratap Singh[1]*

Supplementary Table S1. Screening of antimicrobial activity of endophytic actinobacteria and their biosynthetic genes

Sl No.	Antimicrobial activity (Zone of inhibition in mm ± SE) *									Biosynthetic Genes		
	Isolate No	*S. aureus*	*B. subtilis*	*P. aeruginosa*	*E. coli*	*C. albicans*	*F. proliferatum*	*F. oxysporum*	*F. oxy. ciceri*	PKSI	PKSII	NRPS
1	BPSAC2	0.00±0.00	2.2±0.2	0.00±0.00	4.0± 0.27	0.00±0.00	32.4±0.25	0.00±0.00	0.00±0.00	-	-	-
2	BPSAC7	2.4±0.5	0.00±0.00	5.3±0.11	4.1±0.27	0.00±0.00	0.00±0.00	0.00±0.00	0.00±0.00	-	-	-
3	BPSAC12	0.00±0.00	0.00±0.00	4.6± 0.11	5.3±0.1	0.00±0.00	41.2±0.27	0.00±0.00	0.00±0.00	-	-	-
4	BPSAC14	0.00±0.00	0.00±0.00	0.00±0.00	10.2±0.05	0.00±0.00	35.7±0.3	0.00±0.00	0.00±0.00	-	-	-
5	BPSAC16	0.00±0.00	0.00±0.00	0.00±0.00	5.3±0.1	0.00±0.00	0.00±0.00	0.00±0.00	0.00±0.00	-	-	-
6	BPSAC20	0.00±0.00	0.00±0.00	0.00±0.00	5.6±0.2	0.00±0.00	35.4±0.15	0.00±0.00	0.00±0.00	-	-	-
7	BPSAC24	0.00±0.00	4.2±0.3	0.00±0.00	4.8±0.11	0.00±0.00	0.00±0.00	0.00±0.00	0.00±0.00	-	-	-
8	BPSAC30	0.00±0.00	0.00±0.00	0.00±0.00	3.8±0.11	0.00±0.00	0.00±0.00	0.00±0.00	0.00±0.00	-	-	-
9	BPSAC34	0.00±0.00	6.5±0.65	0.00±0.00	6.1±0.25	0.00±0.00	0.00±0.00	0.00±0.00	0.00±0.00	-	-	-
10	BPSAC35	0.00±0.00	3.5±0.27	0.00±0.00	3.6±0.3	0.00±0.00	0.00±0.00	0.00±0.00	39.2±0.11	-	-	-
11	BPSAC39	0.00±0.00	0.00±0.00	0.00±0.00	8.4±0.23	0.00±0.00	0.00±0.00	0.00±0.00	0.00±0.00	-	-	-
12	BPSAC41	0.00±0.00	5.4±0.05	0.00±0.00	2.1±0.11	6.5±0.65	0.00±0.00	0.00±0.00	0.00±0.00	-	-	-
13	BPSAC44	0.00±0.00	0.00±0.00	0.00±0.00	3.8±0.17	0.00±0.00	0.00±0.00	34.9±0.05	0.00±0.00	-	-	-
14	BPSAC45	0.00±0.00	0.00±0.00	0.00±0.00	10.2±0.05	0.00±0.00	0.00±0.00	36.5±0.3	41.4±0.42	-	-	-
15	BPSAC48	0.00±0.00	0.00±0.00	0.00±0.00	2.8 ±0.6	0.00±0.00	0.00±0.00	51.4±0.35	0.00±0.00	-	-	-
16	BPSAC50	0.00±0.00	3.1 ±0.05	0.00±0.00	5.3±0.3	0.00±0.00	0.00±0.00	37.3±0.22	0.00±0.00	-	-	-
17	BPSAC54	0.00±0.00	0.00±0.00	0.00±0.00	3.6±0.4	0.00±0.00	43.9±0.23	0.00±0.00	0.00±0.00	-	-	-
18	BPSAC56	3.1±0.17	0.00±0.00	0.00±0.00	4.5±0.11	0.00±0.00	0.00±0.00	0.00±0.00	0.00±0.00	-	-	-
19	BPSAC58	0.00±0.00	3.4 ±0.27	0.00±0.00	2.4±0.05	0.00±0.00	0.00±0.00	0.00±0.00	0.00±0.00	-	-	-
20	BPSAC60	0.00±0.00	0.00±0.00	0.00±0.00	4.4±0.05	0.00±0.00	52.4±0.65	48.2±0.2	0.00±0.00	-	-	-
21	BPSAC62	0.00±0.00	2.7±0.05	0.00±0.00	5.1±0.05	0.00±0.00	0.00±0.00	0.00±0.00	34.2±0.37	-	-	-

22	BPSAC63	0.00±0.00	0.00±0.00	0.00±0.00	2.5±0.11	0.00±0.00	0.00±0.00	0.00±0.00	37.4±0.52	-	-	-
23	BPSAC64	5.6±0.17	0.00±0.00	0.00±0.00	6.3± 0.3	0.00±0.00	41.2±0.36	0.00±0.00	0.00±0.00	-	-	-
24	BPSAC65	0.00±0.00	4.4±0.05	0.00±0.00	5.3±0.45	4.4±0.05	0.00±0.00	0.00±0.00	0.00±0.00	-	-	-
25	BPSAC67	7.1±0.05	0.00±0.00	4.6±0.25	6.4±0.05	0.00±0.00	34.3±0.12	0.00±0.00	0.00±0.00	-	+	+
26	BPSAC68	2.5±0.17	0.00±0.00	0.00±0.00	8.6±0.05	4.4±0.05	0.00±0.00	0.00±0.00	0.00±0.00	-	-	-
27	BPSAC69	0.00±0.00	0.00±0.00	0.00±0.00	3.9±0.05	0.00±0.00	0.00±0.00	0.00±0.00	0.00±0.00	-	-	-
28	BPSAC71	0.00±0.00	0.00±0.00	0.00±0.00	1.9±0.6	0.00±0.00	0.00±0.00	50.4±0.05	0.00±0.00	-	-	-
29	BPSAC73	0.00±0.00	3.1 ±0.05	3.1 ±0.05	4.4±0.05	4.4±0.05	0.00±0.00	0.00±0.00	0.00±0.00	-	-	-
30	BPSAC74	7.4±0.17	0.00±0.00	2.3±0.2	2.6±0.05	3.6±0.05	47.4±0.45	0.00±0.00	34.9±0.42	-	-	-
31	BPSAC76	0.00±0.00	0.00±0.00	0.00±0.00	4.8±0.05	0.00±0.00	0.00±0.00	0.00±0.00	0.00±0.00	-	-	-
32	BPSAC77	12.6±0.05	11.2±0.32	10.4±0.37	11.7±0.6	8.9±0.15	42.2±0.3	62.6±0.05	54.8±0.28	+	+	+
33	BPSAC78	1.7±0.1	0.00±0.00	1.5±0.05	4.1±0.56	0.00±0.00	0.00±0.00	0.00±0.00	0.00±0.00	-	-	-
34	BPSAC79	4.6±0.48	5.4±0.11	0.00±0.00	3.8± 0.23	3.1±0.11	0.00±0.00	47.8±0.00	36.2±0.22	-	-	-
35	BPSAC81	0.00±0.00	9.4±0.37	0.00±0.00	3.1±0.05	4.8±0.05	57.2±0.15	0.00±0.00	37.2±0.17	+	+	+
36	BPSAC82	0.00±0.00	6.6±0.27	0.00±0.00	2.6±0.45	0.00±0.00	40.0±0.1	0.00±0.00	0.00±0.00	-	-	-
37	BPSAC84	5.9±0.05	6.5 ±0.37	0.00±0.00	6.9 ±0.15	5.9±0.00	47.5±0.2	32.9±0.05	0.00±0.00	+	+	-
38	BPSAC87	0.00±0.00	0.00±0.00	0.00±0.00	5.1±0.21	3.1±0.15	38.6 ±0.23	51.7±0.17	0.00±0.00	-	+	-
39	BPSAC89	5.1±0.15	0.00±0.00	2.3 ±0.1	3.1±0.21	6.1±0.12	0.00±0.00	45.4±0.1	0.00±0.00	-	-	-
40	BPSAC91	9.7±0.15	6.7±0.27	4.2±0.3	10.1±0.32	2.1±0.27	35.2±0.3	0.00±0.00	0.00±0.00	+	+	+
41	BPSAC93	3.5±0.05	0.00±0.00	6.7±0.27	3.8± 0.23	3.9±0.05	0.00±0.00	39.2±0.37	34.1±0.63	+	+	+
42	BPSAC94	0.00±0.00	0.00±0.00	8.2±0.27	4.1±0.05	0.00±0.00	0.00±0.00	0.00±0.00	0.00±0.00	-	-	-
43	BPSAC98	3.1±0.21	2.9±0.05	0.00±0.00	5.7±0.1	4.5±0.15	0.00±0.00	49.3±0.1	0.00±0.00	-	-	+
44	BPSAC99	5.5±0.3	0.00±0.00	2.9±0.2	6.3±0.3	7.8±0.20	44.5±0.05	47.5±0.05	0.00±0.00	+	+	+
45	BPSAC100	0.00±0.00	0.00±0.00	0.00±0.00	5.4±0.15	0.00±0.00	0.00±0.00	0.00±0.00	0.00±0.00	-	-	-
46	BPSAC101	11.2±0.12	11.6±0.3	9.6±0.27	12.0±0.5	8.1±0.15	41.4±0.37	57.4±0.35	52.4±0.05	+	+	+
47	BPSAC102	0.00±0.00	0.00±0.00	0.00±0.00	6.9±0.05	0.00±0.00	0.00±0.00	0.00±0.00	0.00±0.00	-	-	-
48	BPSAC103	0.00±0.00	3.4±0.3	4.6±0.27	9.2±0.3	2.1±0.20	36.4±0.05	0.00±0.00	0.00±0.00	-	+	+
49	BPSAC104	5.9±0.27	5.8±0.32	8.5±0.27	6.6 ±0.05	3.6±0.37	48.9±0.2	45.8±0.15	0.00±0.00	-	+	+
50	BPSAC110	4.8±0.15	6.1±0.1	8.6±0.65	10.1±0.37	0.00±0.00	55.7±0.05	0.00±0.00	38.5±0.4	+	+	-
51	BPSAC111	5.6±0.5	3.8±0.05	0.00±0.00	5.1±0.15	0.00±0.00	0.00±0.00	0.00±0.00	0.00±0.00	-	-	-
52	BPSAC113	0.00±0.00	0.00±0.00	0.00±0.00	2.9±0.37	0.00±0.00	0.00±0.00	33.5±0.52	39.7±0.32	-	-	-
53	BPSAC114	4.4±0.2	6.3±0.3	0.00±0.00	4.2 ±0.65	4.8±0.15	45.1±0.27	49.7±0.05	0.00±0.00	+	+	+
54	BPSAC115	0.00±0.00	0.00±0.00	0.00±0.00	3.8± 0.23	4.8±0.00	0.00±0.00	44.3±0.05	48.7 ±0.05	+	+	+
55	BPSAC119	0.00±0.00	0.00±0.00	0.00±0.00	8.6±0.6	6.6 ±0.15	0.00±0.00	0.00±0.00	0.00±0.00	-	-	-
56	BPSAC120	7.1±0.2	3.6±0.05	0.00±0.00	5.1 ±0.05	5.0±0.05	58.3±0.2	0.00±0.00	0.00±0.00	+	+	+
57	BPSAC123	0.00±0.00	2.8±0.3	6.4 ±0.05	3.8± 0.23	2.1±0.00	0.00±0.00	47.5±0.1	0.00±0.00	-	+	+
58	BPSAC124	0.00±0.00	2.4±0.37	0.00±0.00	5.6±0.6	5.5±0.3	0.00±0.00	0.00±0.00	0.00±0.00	-	-	-
59	BPSAC126	10.1±0.15	6.2±0.3	3.2±0.11	5.1±0.42	5.0±0.25	38.2±0.17	0.00±0.00	44.2±0.28	+	+	+
60	BPSAC128	5.8±0.15	5.1±0.05	7.5 ±0.27	6.8±0.32	2.8±0.17	52.2±0.27	51.7±0.52	0.00±0.00	+	-	+

61	BPSAC129	3.9±0.2	0.00±0.00	6.2±0.2	3.8± 0.23	0.00±0.00	0.00±0.00	33.0±0.45	0.00±0.00	-	+	-
62	BPSAC130	0.00±0.00	9.3±0.2	2.0±0.2	4.6±0.3	0.00±0.00	0.00±0.00	0.00±0.00	0.00±0.00	-	-	-
63	BPSAC131	0.00±0.00	10.1±0.15	4.5±0.27	9.1±0.05	0.00±0.00	0.00±0.00	34.3±0.42	49.5±0.17	-	-	-
64	BPSAC134	0.00±0.00	2.4 ±0.2	0.00±0.00	4.3±0.2	0.00±0.00	0.00±0.00	0.00±0.00	0.00±0.00	-	-	-
65	BPSAC137	0.00±0.00	0.00±0.00	3.8±0.12	5.1±0.05	0.00±0.00	0.00±0.00	52.1±0.3	0.00±0.00	-	-	-
66	BPSAC139	2.9±0.1	0.00±0.00	0.00±0.00	6.4±0.28	0.00±0.00	0.00±0.00	0.00±0.00	0.00±0.00	-	-	-
67	BPSAC140	3.7±0.27	9.1±0.5	0.00±0.00	6.5±0.45	2.8±0.05	52.7±0.37	0.00±0.00	0.00±0.00	+	-	-
68	BPSAC141	0.00±0.00	0.00±0.00	0.00±0.00	3.8± 0.23	5.6±0.05	32.1±0.28	44.8±0.05	0.00±0.00	-	+	-
69	BPSAC142	0.00±0.00	8.3±0.2	0.00±0.00	3.1±0.3	0.00±0.00	0.00±0.00	0.00±0.00	0.00±0.00	-	-	-
70	BPSAC143	0.00±0.00	2.6±0.1	0.00±0.00	3.8± 0.23	2.1±0.15	43.9±0.05	0.00±0.00	0.00±0.00	-	+	-
71	BPSAC144	8.1±0.15	10.2±0.3	2.6±0.11	4.9±0.42	5.6±0.30	38.7±0.27	0.00±0.00	0.00±0.00	-	+	+
72	BPSAC145	0.00±0.00	0.00±0.00	5.4±0.32	3.8± 0.23	4.3±0.25	0.00±0.00	33.4±0.2	0.00±0.00	+	+	+
73	BPSAC146	0.00±0.00	0.00±0.00	0.00±0.00	5.1±0.21	0.00±0.00	0.00±0.00	0.00±0.00	37.2±0.1	-	-	-
74	BPSAC147	10.8±0.2	10.2±0.3	9.4±0.2	13.2±0.6	8.1±0.17	45.6±0.05	52.1±0.5	49.2±0.15	+	+	+
75	BPSAC150	0.00±0.00	0.00±0.00	6.5 ±0.32	3.8± 0.23	2.4±0.20	0.00±0.00	47.3±0.2	0.00±0.00	-	+	-
76	BPSAC151	0.00±0.00	0.00±0.00	0.00±0.00	2.9±0.05	0.00±0.00	0.00±0.00	0.00±0.00	0.00±0.00	-	-	-
77	BPSAC152	0.00±0.00	7.4±0.37	3.0±0.22	3.6±0.6	0.00±0.00	0.00±0.00	0.00±0.00	0.00±0.00	-	-	-
78	BPSAC153	0.00±0.00	4.2±0.42	3.5±0.65	5.1± 0.15	0.00±0.00	0.00±0.00	0.00±0.00	33.1±0.3	-	+	+
79	BPSAC154	7.1±0.17	7.8±0.15	2.4±0.37	3.8± 0.23	4.8±0.10	32.3±0.2	34.8±0.2	0.00±0.00	-	+	-
80	BPSAC155	0.00±0.00	4.6±0.15	0.00±0.00	7.8±0.4	0.00±0.00	0.00±0.00	0.00±0.00	0.00±0.00	-	-	-
81	BPSAC156	0.00±0.00	5.7±0.05	5.3±0.15	11.2±0.35	6.6±0.05	38.4±0.25	0.00±0.00	0.00±0.00	-	+	+
82	BPSAC157	9.4±0.17	5.1±0.3	0.00±0.00	4.6±0.05	0.00±0.00	0.00±0.00	0.00±0.00	0.00±0.00	-	-	-
83	BPSAC158	7.3±0.42	0.00±0.00	6.8 ±0.52	3.8± 0.23	0.00±0.00	0.00±0.00	35.1±0.11	0.00±0.00	-	+	-
84	BPSAC159	0.00±0.00	0.00±0.00	0.00±0.00	7.5±0.2	3.4±0.37	0.00±0.00	0.00±0.00	0.00±0.00	-	-	-
85	BPSAC161	0.00±0.00	0.00±0.00	3.1±0.15	5.8 ±0.35	7.8±0.05	0.00±0.00	37.1±0.2	38.6±0.22	-	-	-
86	BPSAC162	0.00±0.00	0.00±0.00	0.00±0.00	3.1 ±0.2	0.00±0.00	44.9±0.37	0.00±0.00	0.00±0.00	-	-	-
87	BPSAC166	0.00±0.00	6.4±0.17	2.0±0.21	2.2±0.37	4.8±0.15	58.2±0.15	0.00±0.00	0.00±0.00	-	+	-
88	BPSAC167	0.00±0.00	0.00±0.00	0.00±0.00	3.6±0.32	3.4±0.37	0.00±0.00	0.00±0.00	0.00±0.00	-	-	-
89	BPSAC168	0.00±0.00	0.00±0.00	0.00±0.00	6.2±0.37	0.00±0.00	34.3±0.05	43.0±0.15	0.00±0.00	-	-	-
90	BPSAC169	0.00±0.00	0.00±0.00	2.8±0.05	7.8±0.22	1.9±0.30	0.00±0.00	0.00±0.00	0.00±0.00	-	-	-
91	DBT101	0.00±0.00	5.6±0.3	3.0±0.2	4.6±0.37	0.00±0.00	40.7±0.35	0.00±0.00	0.00±0.00	-	-	-
92	DBT102	0.00±0.00	3.8±0.37	0.00±0.00	9.8±0.17	0.00±0.00	54.5±0.15	0.00±0.00	49.1±0.11	-	-	-
93	DBT103	3.5 ±0.56	0.00±0.00	4.7±0.5	3.1 ±0.56	0.00±0.00	0.00±0.00	0.00±0.00	34.3±0.05	-	-	-
94	DBT104	0.00±0.00	4.2±0.02	5.4±0.15	5.5± 0.20	3.7±0.30	0.00±0.00	0.00±0.00	0.00±0.00	-	-	-
95	DBT105	0.00±0.00	8.1±0.37	4.8±0.11	5.3±0.05	0.00±0.00	57.1±0.23	0.00±0.00	0.00±0.00	-	-	-
96	DBT106	0.00±0.00	6.3 ±0.34	0.00±0.00	4.8± 0.25	0.00±0.00	32.1±0.28	33.4±0.34	0.00±0.00	-	-	-
97	DBT107	0.00±0.00	5.2±0.15	4.3±0.11	3.3±0.27	6.8±0.15	0.00±0.00	0.00±0.00	0.00±0.00	-	-	-
98	DBT108	0.00±0.00	0.00±0.00	0.00±0.00	4.9 ±0.50	3.1±0.25	0.00±0.00	43.9±0.17	48.7 ±0.11	-	-	-
99	DBT111	0.00±0.00	4.3±0.1	0.00±0.00	5.0 ±0.28	5.8±0.05	40.2±0.25	0.00±0.00	0.00±0.00	-	-	-

100	DBT112	5.1±0.1	0.00±0.00	4.9 ±0.17	3.8± 0.23	0.00±0.00	0.00±0.00	48.7±0.11	0.00±0.00	-	-	-
101	DBT113	10.5±0.2	6.6±0.3	3.3±0.2	4.2±0.37	0.00±0.00	0.00±0.00	0.00±0.00	0.00±0.00	-	-	-
102	DBT115	0.00±0.00	0.00±0.00	0.00±0.00	11.3±0.27	0.00±0.00	36.4±0.05	44.2±0.15	32.2±0.2	-	-	-
103	DBT116	0.00±0.00	8.1±0.15	7.2±0.11	3.8± 0.23	0.00±0.00	0.00±0.00	0.00±0.00	44.5±0.50	-	-	-
104	DBT117	3.8±0.42	0.00±0.00	0.00±0.00	3.0± 0.00	0.00±0.00	0.00±0.00	34.4±0.5	40.2±0.28	-	-	-
105	DBT118	6.4±0.17	4.1±0.2	4.0±0.22	3.9±0.05	0.00±0.00	0.00±0.00	0.00±0.00	0.00±0.00	-	-	-
106	DBT119	0.00±0.00	5.6±0.42	9.2±0.2	6.6±0.37	0.00±0.00	53.7±0.35	0.00±0.00	0.00±0.00	-	-	-
107	DBT120	0.00±0.00	0.00±0.00	0.00±0.00	10.0±0.37	4.0±0.15	58.7±0.35	44.0±0.5	0.00±0.00	-	-	-
108	DBT121	0.00±0.00	0.00±0.00	4.3±0.35	5.3± 0.30	3.8±0.05	0.00±0.00	37.6±0.37	0.00±0.00	-	-	-
109	DBT122	0.00±0.00	6.9±0.5	3.9±0.05	4.5± 0.25	4.4±0.05	0.00±0.00	0.00±0.00	0.00±0.00	-	-	-
110	DBT123	0.00±0.00	0.00±0.00	5.5±0.2	4.1±0.17	4.9±0.15	0.00±0.00	35.3±0.42	0.00±0.00	-	-	-
111	DBT124	0.00±0.00	2.4±0.11	0.00±0.00	7.5±0.6	6.8±0.37	56.5±0.05	0.00±0.00	0.00±0.00	-	-	-
112	DBT125	2.1±0.2	4.6±0.2	0.00±0.00	4.5± 0.30	4.1±0.15	0.00±0.00	0.00±0.00	0.00±0.00-	-	-	-
113	BPSAC5	0.00±0.00	0.00±0.00	0.00±0.00	2.6±0.37	0.00±0.00	51.7±0.37	0.00±0.00	0.00±0.00	-	-	-
114	BPSAC13	0.00±0.00	0.00±0.00	3.6±0.11	3.4±0.12	0.00±0.00	55.6±0.5	0.00±0.00	0.00±0.00	-	-	-
115	BPSAC21	4.9±0.05	0.00±0.00	0.00±0.00	6.7±0.37	0.00±0.00	0.00±0.00	0.00±0.00	0.00±0.00	-	-	-
116	BPSAC22	0.00±0.00	0.00±0.00	0.00±0.00	4.1±0.02	0.00±0.00	0.00±0.00	0.00±0.00	0.00±0.00	-	-	-
117	BPSAC26	0.00±0.00	2.5±0.28	0.00±0.00	3.4±0.12	4.7±0.1 1	0.00±0.00	0.00±0.00	0.00±0.00	-	-	-
118	BPSAC29	0.00±0.00	0.00±0.00	0.00±0.00	4.7±0.36	0.00±0.00	0.00±0.00	46.6±0.22	0.00±0.00	-	-	-
119	BPSAC32	5.3±0.27	0.00±0.00	0.00±0.00	4.0±0.15	0.00±0.00	0.00±0.00	0.00±0.00	46.4±0.12	-	-	-
120	BPSAC37	0.00±0.00	0.00±0.00	0.00±0.00	2.1±0.17	4.1±0.1 1	0.00±0.00	0.00±0.00	0.00±0.00	-	-	-
121	BPSAC38	0.00±0.00	3.3±0.57	0.00±0.00	7.8±0.65	6.7±0.1 5	0.00±0.00	0.00±0.00	0.00±0.00	-	-	-
122	BPSAC43	0.00±0.00	0.00±0.00	3.4±0.12	7.7 ±0.28	3.2±0.12	0.00±0.00	0.00±0.00	0.00±0.00	-	-	-
123	BPSAC46	0.00±0.00	0.00±0.00	7.2±0.2	3.6±0.37	0.00±0.00	0.00±0.00	0.00±0.00	34.2±0.37	-	-	-
124	BPSAC47	0.00±0.00	0.00±0.00	0.00±0.00	9.6±0.65	0.00±0.00	0.00±0.00	0.00±0.00	0.00±0.00	-	-	-
125	BPSAC49	2.7±0.1 1	5.2±0.05	0.00±0.00	7.3±0.5	0.00±0.00	0.00±0.00	0.00±0.00	0.00±0.00	-	-	-
126	BPSAC51	4.3±0.37	0.00±0.00	8.0±0.1	5.1±0.22	0.00±0.00	0.00±0.00	0.00±0.00	0.00±0.00	-	-	-
127	BPSAC52	0.00±0.00	0.00±0.00	0.00±0.00	6.2±0.05	0.00±0.00	0.00±0.00	0.00±0.00	0.00±0.00	-	-	-
128	BPSAC53	0.00±0.00	0.00±0.00	0.00±0.00	3.7±0.56	0.00±0.00	41.7±0.01	0.00±0.00	0.00±0.00	-	-	-
129	BPSAC55	0.00±0.00	0.00±0.00	0.00±0.00	7.4±0.1	0.00±0.00	0.00±0.00	34.2±0.52	0.00±0.00	-	-	-
130	BPSAC57	0.00±0.00	6.9±0.27	4.3±0.45	2.3±0.56	0.00±0.00	0.00±0.00	0.00±0.00	0.00±0.00	-	-	-
131	BPSAC59	0.00±0.00	0.00±0.00	0.00±0.00	6.2±0.05	4.4±0.12	0.00±0.00	44.6±0.17	0.00±0.00	-	-	-
132	BPSAC61	0.00±0.00	0.00±0.00	0.00±0.00	8.4±0.37	0.00±0.00	44.8±0.45	37.3±0.11	0.00±0.00	-	-	-
133	BPSAC70	0.00±0.00	0.00±0.00	3.3±0.57	3.6 ±0.05	0.00±0.00	0.00±0.00	0.00±0.00	0.00±0.00	-	-	-
134	BPSAC72	0.00±0.00	0.00±0.00	0.00±0.00	5.4±0.4	0.00±0.00	0.00±0.00	0.00±0.00	0.00±0.00	-	-	-
135	BPSAC75	0.00±0.00	0.00±0.00	0.00±0.00	3.1±0.05	0.00±0.00	0.00±0.00	0.00±0.00	0.00±0.00	-	-	-
136	BPSAC80	0.00±0.00	0.00±0.00	6.6±0.42	3.8± 0.23	3.2±0.05	31.4±0.25	0.00±0.00	0.00±0.00	-	-	-
137	BPSAC83	3.5±0.42	0.00±0.00	0.00±0.00	7.5±0.5	0.00±0.00	0.00±0.00	32.7±0.36	0.00±0.00	-	-	-
138	BPSAC85	0.00±0.00	9.8±0.27	0.00±0.00	8.1±0.3	4.8±0.15	54.7±0.05	0.00±0.00	46.2±0.27	+	-	-

139	BPSAC86	5.1±0.2	0.00±0.00	6.9±0.23	4.6±0.2	5.6±0.05	0.00±0.00	0.00±0.00	0.00±0.00	+	+	-
140	BPSAC88	0.00±0.00	5.9±0.2	0.00±0.00	7.9±0.4	0.00±0.00	0.00±0.00	0.00±0.00	0.00±0.00	-	-	-
141	BPSAC90	0.00±0.00	0.00±0.00	3.2±0.23	2.7±0.6	0.00±0.00	45.1 ±0.1	0.00±0.00	0.00±0.00	-	-	-
142	BPSAC92	0.00±0.00	6.7±0.27	5.7±0.05	5.2±0.5	3.1±0.03	49.4±0.05	0.00±0.00	0.00±0.00	-	+	-
143	BPSAC95	0.00±0.00	9.1±0.15	8.3±0.2	8.0±0.05	7.4±0.15	37.8±0.15	0.00±0.00	0.00±0.00	-	-	-
144	BPSAC96	3.1±0.17	3.6±0.5	0.00±0.00	5.7±0.17	4.8±0.30	51.2±0.3	0.00±0.00	0.00±0.00	-	-	-
145	BPSAC97	0.00±0.00	0.00±0.00	0.00±0.00	4.0±0.2	0.00±0.00	0.00±0.00	0.00±0.00	0.00±0.00	-	-	-
146	BPSAC105	0.00±0.00	0.00±0.00	0.00±0.00	3.6±0.1	0.00±0.00	0.00±0.00	0.00±0.00	0.00±0.00	-	-	-
147	BPSAC106	0.00±0.00	7.4±0.05	0.00±0.00	2.4±0.27	5.9±0.15	0.00±0.00	41.2±0.05	0.00±0.00	+	+	+
148	BPSAC107	0.00±0.00	0.00±0.00	7.6±0.22	3.8± 0.23	2.8±0.25	0.00±0.00	0.00±0.00	32.8 ±0.05	+	+	+
149	BPSAC108	3.1±0.2	0.00±0.00	4.3±0.15	5.2±0.3	0.00±0.00	0.00±0.00	0.00±0.00	0.00±0.00	-	-	-
150	BPSAC109	4.6±0.1	0.00±0.00	7.5±0.13	7.1± 0.20	0.00±0.00	52.0±0.00	0.00±0.00	44.6±0.11	-	+	-
151	BPSAC112	3.1±0.15	8.1±0.65	4.7±0.5	8.0±0.1	4.5±0.37	44.7±0.45	52.0±0.2	0.00±0.00	+	+	+
152	BPSAC116	0.00±0.00	0.00±0.00	0.00±0.00	5.2±0.37	5.7±0.25	38.5±0.15	35.4±0.05	0.00±0.00	+	+	+
153	BPSAC117	0.00±0.00	0.00±0.00	0.00±0.00	3.1±0.05	0.00±0.00	0.00±0.00	38.1±0.45	39.7±0.36	-	-	-
154	BPSAC118	0.00±0.00	4.3±0.45	0.00±0.00	5.9±0.25	7.0±0.05	0.00±0.00	40.8±0.34	0.00±0.00	-	-	+
155	BPSAC121	11.1±0.5	10.6±0.5	9.4±0.05	12.8±0.00	9.8±0.15	59.2±0.5	63.7±0.05	51.6±0.00	+	+	+
156	BPSAC122	0.00±0.00	4.9±0.37	0.00±0.00	4.0±0.38	0.00±0.00	0.00±0.00	0.00±0.00	0.00±0.00	-	-	-
157	BPSAC125	0.00±0.00	3.4±0.3	0.00±0.00	3.8± 0.23	4.8±0.02	0.00±0.00	46.0±0.1	0.00±0.00	-	+	+
158	BPSAC127	4.60±0.15	0.00±0.00	7.9±0.35	5.4±0.37	0.00±0.00	0.00±0.00	0.00±0.00	0.00±0.00	-	-	-
159	BPSAC132	0.0±0.05	0.00±0.00	0.00±0.00	8.2±0.27	0.00±0.00	41.4±0.22	46.6±0.42	0.00±0.00	-	-	-
160	BPSAC133	10.5±0.2	0.00±0.00	0.00±0.00	4.1±0.37	5.2±0.15	55.7±0.35	0.00±0.00	0.00±0.00	-	+	-
161	BPSAC135	0.00±0.00	5.7±0.5	6.2±0.65	3.8± 0.25	2.8±0.30	0.00±0.00	0.00±0.00	0.00±0.00	+	-	-
162	BPSAC136	8.7±0.37	10.1±0.15	4.5±0.27	9.0±0.05	4.1±0.37	38.4±0.15	34.3±0.42	0.00±0.00	+	+	+
163	BPSAC138	0.00±0.00	1.8±0.21	5.7±0.11	7.4±0.37	7.1±0.15	0.00±0.00	0.00±0.00	0.00±0.00	+	+	+
164	BPSAC148	3.1±0.11	0.00±0.00	0.00±0.00	6.8±0.17	0.00±0.00	0.00±0.00	0.00±0.00	48.1±0.5	-	-	-
165	BPSAC149	4.5±0.27	0.00±0.00	0.00±0.00	9.7±0.45	3.9±0.25	0.00±0.00	44.7±0.45	0.00±0.00	-	+	+
166	BPSAC160	0.00±0.00	0.00±0.00	0.00±0.00	8.2±0.28	0.00±0.00	0.00±0.00	39.0±0.05	0.00±0.00	-	-	-
167	BPSAC163	4.6±0.05	0.00±0.00	0.00±0.00	7.7±0.05	5.0±0.37	51.4±0.11	0.00±0.00	00.00±0.00	-	-	+
168	BPSAC164	10.4±0.17	4.4±0.2	3.8±0.05	5.2±0.65	0.00±0.00	33.6±0.27	41.4±0.5	47.6±0.05	-	+	-
169	BPSAC165	0.00±0.00	0.00±0.00	0.00±0.00	8.1±0.11	3.1±0.11	0.00±0.00	0.00±0.00	0.00±0.00	-	-	-

(+) & (-) indicates the presence and absence of biosynthetic genes

Supplementary Table S2. Identical similarity of 16S rRNA gene with endophytic actinobacteria

Strain	Isolate name	Accession No	Identity	Similarity	Biosynthetic gene Accession No		
					PKSI	PKSII	NRPS
BPSAC67	*Actinobacterium*	KJ914905	*Streptomyces olivaceus* type strain NBRC 12805	98.7%	-	KU879306	KU899075
BPSAC73	*Kocuria* sp.	KJ914911	*Kocuria rhizophila* type strain DSM 11926	99.2%	-	-	-
BPSAC74	*Microbacterium testaceum*	KP128838	*Microbacterium testaceum* type strain DSM 20166	98.2%	-	-	-
BPSAC77	*Streptomyces olivaceus*	KP128839	*Streptomyces olivaceus* type strain NBRC 12805	99.4%	KU956018	KT187712	KU899056
BPSAC79	*Microbacterium arborescens*	KP264911	*Microbacterium arborescens* type strain DSM 20754	100%	-	-	-
BPSAC81	*Streptomyces atroolivaceus*	KP128841	*Streptomyces atroolivaceus* type strain LMG 19306	99.9%	KU956019	KU879327	KU899057
BPSAC84	*Micromonospora* sp.	KP128842	*Micromonospora aurantiaca* type strain ATCC 27029	99.9%	KU956020	KT187713	-
BPSAC87	*Streptomyces* sp.	KP128845	*Streptomyces olivicoloratus* type strain T13	98.0%	-	KT187714	-
BPSAC89	*Micrococcus luteus*	KP128846	*Micrococcus luteus* type strain NCTC 2665	99.1%	-	-	-
BPSAC91	*Streptomyces* sp.	KP128847	*Streptomyces albidoflavus* type strain DSM 40455	99.9%	KU956021	KU879328	KU899058

BPSAC93	*Streptomyces* sp.	KP128849	*Streptomyces cellulosae* type strain NBRC 13027	98.8%	KU956022	KT187715	KU899059
BPSAC98	*Nocardiopsis synnemataformans*	KP128852	*Nocardiopsis synnemataformans* type strain DSM 44143	99.7%	-	-	KU899060
BPSAC99	*Streptomyces thermocarboxydus*	KP128853	*Streptomyces thermocarboxydus* type strain DSM 44293	99.4%	KU956023	KU879295	KU899061
BPSAC101	*Streptomyces* sp.	KP128854	*Streptomyces marokkonensis* type strain Ap1	98.7%	KU956024	KT187716	KU899062
BPSAC103	*Streptomyces* sp.	KP128855	*Streptomyces marokkonensis* type strain Ap1	99.7%	-	KT187717	KU899063
BPSAC104	*Streptomyces* sp.	KP128856	*Streptomyces malachitofuscus* type strain NBRC 13059	98.2%	-	-	KU899064
BPSAC110	*Streptomyces* sp.	KP128860	*Streptomyces cellulosae* type strain NBRC 13027	99.9%	KU956025	KU879296	-
BPSAC114	*Streptomyces cellulosae*	KP264912	*Streptomyces cellulosae* type strain NBRC 13027	99.8%	KU956026	KU879304	KU899073
BPSAC115	*Streptomyces* sp.	KP128862	*Streptomyces marokkonensis* type strain Ap1	99.9%	KU956027	-	KU899065
BPSAC120	*Streptomyces* sp.	KP128865	*Streptomyces malachitofuscus* type strain NBRC 13059	98.1%	KU956028	KU879297	KU899066
BPSAC123	*Streptomyces rutgersensis*	KP128867	*Streptomyces rutgersensis* type strain NBRC 12819	99.0%	-	KU879317	KU899067
BPSAC126	*Streptomyces mutabilis*	KP264913	*Streptomyces mutabilis* type strain	100%	KU956029	KU879305	KU899074

			NBRC12800				
BPSAC128	*Leifsonia xyli*	KP128869	*Leifsonia xyli subsp. cynodontis* type strain JCM9733	100%	KX894556	-	KX894557
BPSAC129	*Streptomyces* sp.	KP128870	*Streptomyces olivaceus* type strain NBRC 12805	98.8%	-	KU879298	-
BPSAC131	*Microbacterium* sp.	KP264914	*Microbacterium testaceum* type strain DSM 20166	99.5%	-	-	-
BPSAC140	*Brevibacterium* sp.	KP128875	*Brevibacterium casei* type strain NCDO2048	98.2%	KU956030	-	-
BPSAC141	*Streptomyces* sp.	KP128876	*Streptomyces albidoflavus* type strain DSM 40455	98.5%	-	KU879299	-
BPSAC143	*Streptomyces mutabilis*	KP128877	*Streptomyces mutabilis* type strain NBRC12800	100%	-	KU879300	-
BPSAC144	*Streptomyces olivaceus*	KP128878	*Streptomyces olivaceus* type strain NBRC 12805	100%	-	KU879301	KU899068
BPSAC145	*Actinobacterium*	KP128879	*Streptomyces olivaceus* type strain NBRC 12805	98.7%	KU956031	KU879318	KU899069
BPSAC147	*Streptomyces thermocarboxydus*	KP128880	*Streptomyces thermocarboxydus* type strain DSM 44293	100%	KU956032	KU879302	KU899070
BPSAC150	*Streptomyces mutabilis*	KP128882	*Streptomyces mutabilis* type strain NBRC12800	100%	-	KU879319	-
BPSAC153	*Streptomyces* sp.	KP128883	*Streptomyces olivaceus* type strain NBRC 12805	99.9%	-	KU879303	KU899071

BPSAC154	*Microbacterium* sp.	KP128884	*Microbacterium testaceum* type strain DSM 20166	98.9%	-	KU879320	-
BPSAC156	*Streptomyces steffisburgensis*	KP128885	*Streptomyces marokkonensis* type strain Ap1	98.1%	-	KU879321	KU899072
BPSAC158	*Pseudonocardia* sp.	KP128886	*Pseudonocardia carboxydivorans* type strain Y8	100%	-	KU879322	-
BPSAC161	*Actinomycetales bacterium*	KP128887	*Streptomyces albidoflavus* type strain DSM 40455	98.6%	-	-	-
BPSAC166	*Micrococcus yunnanensis*	KP128890	*Micrococcus yunnanensis* type strain YIM 65004	100%	-	KU879329	-
DBT101	*Streptomyces* sp.	KU195388	*Streptomyces albidoflavus* type strain DSM 40455	98.4%	-	-	-
DBT102	*Amycolatopsis* sp.	KU195389	*Amycolatopsis rifamycinica* type strain DSM 46095	98.1%	-	-	-
DBT103	*Amycolatopsis* sp.	KU195390	*Amycolatopsis rifamycinica* type strain DSM 46095	100%	-	-	-
DBT104	*Streptomyces* sp.	KU195391	*Streptomyces xiamenensis* type strain MCCC 1A01550	98.9%	-	-	-
DBT105	*Amycolatopsis balhimycina*	KU195392	*Amycolatopsis balhimycina* type strain FH 1894	98.7%	-	-	-
DBT106	*Streptomyces* sp.	KU195393	*Streptomyces albidoflavus* type strain DSM 40455	98.4%	-	-	-
DBT107	*Streptomyces* sp.	KU195394	*Streptomyces albidoflavus* type	99.7%	-	-	-

			strain DSM 40455				
DBT108	*Streptomyces* sp.	KU195395	*Streptomyces xiamenensis* type strain MCCC 1A01550	98.7%	-	-	-
DBT111	*Streptomyces thermocarboxydus*	KU195398	*Streptomyces thermocarboxydus* type strain DSM 44293	99.9%	-	-	-
DBT112	*Actinomycete*	KU195399	*Streptomyces albidoflavus* type strain DSM 40455	98.7%	-	-	-
DBT113	*Microbacterium* sp.	KU195400	*Microbacterium paraoxydans* type strain CF36	98.3%	-	-	-
DBT115	*Streptomyces* sp.	KU195402	*Streptomyces olivaceus* type strain NBRC 12805	99.5%	-	-	-
DBT116	*Nocardiopsis* sp.	KU195403	*Nocardiopsis synnemataformans* type strain DSM 44143	100%	-	-	-
DBT117	*Tsukamurella tyrosinosolvens*	KU195404	*Tsukamurella tyrosinosolvens* type strain IMMIB D-1397	100%	-	-	-
DBT118	*Streptomyces* sp.	KU195405	*Streptomyces albidoflavus* type strain DSM 40455	98.9%	-	--	-
DBT119	*Streptomyces thermocarboxydus*	KU195406	*Streptomyces thermocarboxydus* type strain DSM 44293	99.8%	-	-	-
DBT120	*Streptomyces* sp.	KU195407	*Streptomyces albidoflavus* type strain DSM 40455	99.4%	-	-	-
DBT121	*Streptomyces* sp.	KU195408	*Streptomyces albidoflavus* type strain DSM 40455	98.9%	-	-	-

DBT122	*Streptomyces* sp.	KU195409	*Streptomyces albidoflavus* type strain DSM 40455	99.0%	-	-	-
DBT123	*Streptomyces* sp.	KU195410	*Streptomyces thermocarboxydus* type strain DSM 44293	100%	-	-	-
DBT124	*Streptomyces pactum*	KU195411	*Streptomyces pactum* type strain NBRC 13433	98.7%	-	-	-
DBT125	*Promicromonospora* sp.	KU195412	*Promicromonospora sukumoe* type strain IFO14650	100%	-	-	-
BPSAC80	*Microbacterium* sp.	KP128840	*Microbacterium testaceum* type strain DSM 20166	100%	-	-	-
BPSAC85	*Streptomyces* sp.	KP128843	*Streptomyces rutgersensis* type strain NBRC 12819	98.8%	KU956033	-	-
BPSAC86	*Streptomyces* sp.	KP128844	*Streptomyces albidoflavus* type strain DSM 40455	98.7%	KU956034	KU879323	-
BPSAC92	*Streptomyces cellulosae*	KP128848	*Streptomyces cellulosae* type strain NBRC 13027	100%	-	KU879315	-
BPSAC95	*Kocuria palustris*	KP128850	*Kocuria palustris* type strain DSM 11925	100%	-	-	-
BPSAC96	*Nocardiopsis* sp.	KP128851	*Nocardiopsis dassonvillei* subsp. albirubida type strain DSM 40465	100%	-	-	-
BPSAC106	*Micromonospora* sp.	KP128857	*Micromonospora aurantiaca* type strain ATCC 27029	100%	KU956035	KU879307	KU899076
BPSAC107	*Micromonospora* sp.	KP128858	*Micromonospora aurantiaca* type	99.9%	-	KU879308	KU899077

			strain ATCC 27029				
BPSAC109	*Streptomyces* sp.	KP128859	*Streptomyces olivaceus* type strain NBRC12805	100%	-	KU879309	-
BPSAC112	*Streptomyces olivaceus*	KP128861	*Streptomyces olivaceus* type strain NBRC12805	99.9%	KU956036	KU879324	KU899078
BPSAC116	*Actinomycetales bacterium*	KP128863	*Streptomyces malachitofuscus* type strain NBRC 13059	98.9%	KU956037	KU879310	KU899079
BPSAC118	*Streptomyces tempisquensis*	KP128864	*Streptomyces xiamenensis* type strain MCCC 1A01550	99.2%	-	-	KU899080
BPSAC121	*Streptomyces* sp.	KP128866	*Streptomyces olivaceus* type strain NBRC12805	98.7%	KU956038	KU879311	KU899081
BPSAC125	*Kocuria palustris*	KP128868	*Kocuria palustris* type strain DSM 11925	100%	-	KU879312	KU899082
BPSAC133	*Streptomyces thermocarboxydus*	KP128871	*Streptomyces thermocarboxydus* type strain DSM 44293	100%	-	KU879325	-
BPSAC135	*Streptomyces* sp.	KP128872	*Streptomyces erringtonii* type strain I36	98.6%	KU956039	-	-
BPSAC136	*Streptomyces* sp.	KP128873	*Streptomyces thermocarboxydus* type strain DSM 44293	99.5%	KU956040	KU879313	KU899083
BPSAC138	*Streptomyces* sp.	KP128874	*Streptomyces olivaceus* type strain NBRC12805	99.8%	KU956041	KU879326	KU899084
BPSAC149	*Streptomyces* sp.	KP128881	*Streptomyces oliveceus* type strain NBRC12805	100%	-	KU879314	KU899085

BPSAC163	*Saccharopolyspora* sp.	KP128888	*Saccharopolyspora tripterygii* type strain YIM 65359	98.2%	-	-	KU899086
BPSAC164	*Rhodococcus* sp.	KP128889	*Rhodococcus wratislaviensis* type strain NBRC 100605	100%	-	KU879316	-

Supplementary Table S3. Antibiotic sensitivity pattern and plant growth promoting attributes of selected antimicrobial endophytic actinobacterial isolates

Isolate No	Plant Growth Promoting Traits (PGPR)					Antibiotic Sensitivity[$]											
	Phosphate (mm)*	Ammonia (mg/ml)*	IAA (µg/ml)*	Cellulase U/mL*	Amylase U/mL*	V	G	P	N	T	Ne	A	C	E	S	Ny	R
BPSAC67	-	-	21.4±0.20	-	42.4±0.10	I	I	R	S	S	R	R	S	S	R	I	I
BPSAC73	-	-	-	31.2±0.27	-	S	I	R	S	S	R	R	S	S	S	S	S
BPSAC74	5.2±0.15	31.3±0.25	31.2±0.20	52.7±0.10	-	I	R	R	I	S	R	R	S	I	I	R	R
BPSAC77	9.7±0.10	78.8±0.10	52.3±0.10	74.2±0.37	74.8±0.25	I	I	R	R	S	R	R	S	R	R	R	I
BPSAC79	-	16.2±0.20	7.4±0.17	-	-	S	I	R	S	S	I	R	S	S	S	R	I
BPSAC81	-	21.7±0.17	-	-	-	S	I	R	S	S	R	R	S	S	R	S	R
BPSAC84	-	34.2±0.10	21.4±0.25	-	34.2±0.20	I	R	R	S	S	R	R	S	S	I	S	I
BPSAC87	-	-	-	-	-	S	I	R	S	S	I	R	S	S	R	R	S
BPSAC89	-	8.6±0.30	-	-	-	I	S	R	S	S	S	R	S	S	R	I	S
BPSAC91	-	21.7±0.25	27.4±0.17	-	32.6±0.10	S	I	R	S	S	R	R	S	S	S	R	R
BPSAC93	6.2±0.20	14.8±0.20	7.4±0.15	34.2±0.25	-	I	S	R	S	S	S	R	S	S	R	R	I
BPSAC98	-	37.1±0.05	-	-	-	S	I	R	I	S	R	R	S	S	R	S	I
BPSAC99	-	53.6±0.20	36.1±0.15	-	44.8±0.17	I	S	R	S	S	R	R	S	S	S	R	I
BPSAC101	8.4±0.25	74.2±0.17	42.6±0.15	67.5±0.20	48.1±0.15	S	S	R	R	S	R	R	S	R	R	R	I
BPSAC103	-	12.4±0.15	11.2±0.37	-	-	S	S	R	I	S	R	R	S	S	I	S	R
BPSAC104	-	-	-	-	36.7±0.20	I	R	R	S	S	R	R	S	S	R	R	R
BPSAC110	-	49.6±0.27	18.4±0.15	-	-	S	I	R	S	S	R	R	S	S	S	I	R
BPSAC114	4.6±0.15	71.2±0.15	42.6±0.20	39.4±0.17	-	I	S	R	S	S	R	R	S	R	I	R	S
BPSAC115	-	-	-	-	-	S	I	R	S	S	R	R	S	S	R	S	S
BPSAC120	3.8±0.17	46.3±0.20	9.8±0.10	41.2±0.37	-	S	I	R	S	S	R	R	S	S	R	R	I
BPSAC123	-	-	-	-	39.8±0.25	S	I	R	S	S	R	R	S	S	R	S	I
BPSAC126	-	32.5±0.15	11.9±0.37	-	-	S	I	R	S	S	S	R	S	S	S	R	I
BPSAC128	2.8±0.20	12.4±0.10	25.4±0.20	45.2±0.25	32.8±0.20	I	I	R	S	S	R	R	S	I	R	R	I
BPSAC129	-	-	-	-	-	S	I	R	S	S	R	R	S	S	S	S	S
BPSAC131	-	36.3±0.25	24.6±0.21	-	49.8±0.15	I	R	R	I	S	R	R	S	R	I	R	R
BPSAC140	6.1±0.17	52.3±0.37	37.2±0.10	-	44.1±0.37	R	R	R	I	S	R	R	S	I	R	R	I
BPSAC141	-	-	-	-	-	S	I	R	S	S	I	R	S	S	S	R	I
BPSAC143	5.2±0.15	21.7±0.20	21.6±0.20	51.2±0.17	-	S	I	R	S	S	R	R	S	S	R	S	R
BPSAC144	-	24.2±0.10	11.5±0.37	-	-	I	R	R	S	S	R	R	S	S	I	S	I
BPSAC145	6.8±0.17	41.5±0.25	-	-	-	S	I	R	S	S	I	R	S	S	R	R	S
BPSAC147	7.6±0.20	72.6±0.30	39.4±0.15	50.6±0.20	46.2±0.27	R	S	R	R	S	S	R	S	S	R	R	R
BPSAC150	-	-	-	48.2±0.25	-	S	I	R	S	S	R	R	S	S	S	R	R

BPSAC153	-	-	-	-	41.8±0.17	S	S	R	S	S	S	R	S	S	R	R	I
BPSAC154	-	29.1±0.15	16.2±0.10	-	-	S	I	R	I	S	R	R	S	S	R	S	I
BPSAC156	-	33.6±0.20	34.1±0.17	-	-	S	S	R	S	S	R	R	S	S	S	R	I
BPSAC158	2.4±0.10	57.3±0.17	9.2±0.25	42.6±0.10	48.6±0.10	S	S	R	S	S	R	R	S	S	R	R	I
BPSAC161	-	32.4±0.20	38.2±0.30	-	-	S	S	R	I	S	R	R	S	S	I	S	R
BPSAC166	-	-	-	-	37.5±0.25	I	R	R	S	S	R	R	S	S	R	R	R
DBT101	-	-	-	-	-	S	I	R	S	S	R	R	S	S	S	I	R
DBT102	-	64.2±0.10	22.6±0.20	-	45.4±0.17	I	S	R	S	S	R	R	S	S	I	R	S
DBT103	3.1±0.17	30.8±0.17	-	-	34.8±0.25	S	I	R	S	S	R	R	S	S	R	S	S
DBT104	5.6±0.20	46.3±0.25	11.8±0.10	51.0±0.20	32.7±0.30	S	I	R	S	S	R	R	S	S	S	R	I
DBT105	-	-	-	-	-	S	I	R	S	S	R	R	S	S	S	S	I
DBT106	-	12.5±0.15	-	-	33.1±0.17	S	I	R	S	S	S	R	S	S	S	R	I
DBT107	-	-	-	34.2±0.30	52.6±0.20	S	I	R	S	S	R	R	S	S	R	I	I
DBT108	-	-	-	48.6±0.17	-	S	I	R	S	S	R	R	S	S	S	S	S
DBT111	-	27.1±0.15	-	-	50.3±0.25	I	R	R	I	S	R	R	S	S	I	R	R
DBT112	-	-	-	-	34.8±0.20	R	R	R	I	S	R	R	S	I	R	R	I
DBT113	-	-	-	-	-	S	I	R	S	S	I	R	S	S	S	R	I
DBT115	-	51.2±0.25	31.2±0.25	-	-	S	I	R	S	S	R	R	S	S	R	S	R
DBT116	7.1±0.15	35.4±0.15	-	38.2±0.15	-	I	R	R	S	S	R	R	S	S	I	S	I
DBT117	6.3±0.10	40.5±0.25	-	49.5±0.20	-	S	I	R	S	S	I	R	S	S	R	R	S
DBT118	-	-	-	-	-	S	S	R	S	S	S	R	S	S	R	I	S
DBT119	2.6±0.20	11.2±0.10	11.2±0.20	-	45.6±0.10	S	I	R	R	S	R	R	S	S	S	R	R
DBT120	-	-	34.1±0.15	-	42.4±0.15	I	S	R	S	S	S	R	S	S	R	R	I
DBT121	3.4±0.15	17.4±0.30	-	-	-	S	I	R	I	S	R	R	S	S	R	S	I
DBT122	-	-	-	37.1±0.25	38.2±0.17	I	S	R	S	S	R	R	S	S	S	R	I
DBT123	-	-	-	-	39.3±0.20	S	S	R	S	S	R	R	S	S	R	R	I
DBT124	-	-	-	45.2±0.17	-	S	S	R	I	S	R	R	S	S	I	S	R
DBT125	-	28.1±0.37	12.6±0.10	-	-	I	R	R	S	S	R	R	S	S	R	R	R
BPSAC80	-	-	-	-	-	S	I	R	S	S	R	R	S	S	S	I	R
BPSAC85	-	-	-	-	48.1±0.15	S	S	R	S	S	R	R	S	S	I	R	S
BPSAC86	-	-		39.7±0.10	-	S	I	R	S	S	R	R	S	S	R	S	S
BPSAC92	5.8±0.20	32.1±0.20	21.4±0.20	-	37.2±0.27	S	I	R	S	S	R	R	S	S	S	R	I
BPSAC95	-	51.6±0.25	31.2±0.37	-	34.6±0.20	S	I	R	S	S	R	R	S	S	S	S	I
BPSAC96	-	-	-	-	-	S	I	R	S	S	S	R	S	S	S	R	I
BPSAC106	4.7±0.17	12.4±0.15	22.3±0.15	-	-	S	I	R	S	S	R	R	S	S	R	I	I
BPSAC107	-	-	-	-	36.2±0.15	S	I	R	S	S	R	R	S	S	S	S	S
BPSAC109	-	11.4±0.37	-	-	49.7±0.10	I	R	R	I	S	S	R	S	S	I	R	R
BPSAC112	-	47.6±0.25	-	-	51.2±0.37	R	R	R	I	S	S	R	S	I	R	R	I
BPSAC116	-	-	-	-	-	S	I	R	S	S	I	R	S	S	S	R	I

BPSAC118	-	-	-	-	42.5±0.15	S	I	R	S	S	S	R	S	S	R	S	R
BPSAC121	8.2±0.20	82.3±0.15	47.8±0.17	37.8±0.25	50.3±0.20	I	R	R	R	S	R	R	S	S	R	R	I
BPSAC125	-	-	-	44.2±0.37	-	S	I	R	S	S	I	R	S	S	R	R	S
BPSAC133	-	8.6±0.27	11.2±0.10	-	-	I	S	R	S	S	S	R	S	S	R	I	S
BPSAC135	-	-	-	37.5±0.15	48.2±0.15	S	I	R	S	S	S	R	S	S	S	R	R
BPSAC136	-	24.8±0.20	9.2±0.15	-	34.4±0.37	I	S	R	S	S	S	R	S	S	R	R	I
BPSAC138	-	-	-	34.2±0.20	-	S	I	R	S	S	I	R	S	S	R	R	S
BPSAC149	-	-	-	-	32.6±0.17	I	S	R	S	S	S	R	S	S	R	I	S
BPSAC163	-	-	-	-	49.5±0.20	S	I	R	S	S	S	R	S	S	S	R	R
BPSAC164	-	32.3±0.15	-	-	38.8±0.30	S	S	R	S	S	S	R	S	S	R	R	I

*Each value represents a mean ± standard error (SE).

$ Degree of susceptibility: S: sensitive (>10 mm); I: intermediate (5.0-9.9 mm); R: resistant (0.0-4.9 mm).

Supplementary Table S4. Method validation parameters for sixteen reference analytes in four strains

Analytes	Regression Equation	r^2	Linear range (ng/ml)	LOD (ng/ml)	LOQ (ng/ml)	Precision RSD (%)		Stability	Recovery RSD (%)
						Intra-day (n=6)	Inter-day (n=6)	RSD (n = 5)	
Fluconazole	y = 8583.1x + 3165	0.9997	0.5-100	0.10	0.40	0.32	1.04	0.85	1.56
Chloramphenicol	y = 564.79x + 492.15	0.9999	0.5-100	0.13	0.39	0.71	0.88	1.22	2.05
Erythromycin	y = 3178.7x + 725.71	0.9997	0.5-100	0.15	0.45	1.03	2.03	1.09	1.87
Ketoconazole	y = 256.47x - 4471.5	0.9991	10-250	3.50	8.50	1.82	1.05	1.16	1.36
Rifampicin	y = 5680.6x - 4052.3	0.9995	0.5-100	0.10	0.40	0.53	1.37	0.56	1.28
Miconazole	y = 1564.9x + 19304	0.9999	1-100	0.32	0.71	1.29	1.92	0.83	1.81
Catechin	y = 7.01*x* + 0.34	0.9999	1-250	0.15	0.45	0.35	1.01	1.87	0.75
Kaempferol	y = 5.54*x* + 0.32	0.9998	1-250	0.23	0.71	1.05	1.21	2.57	1.05
Chabulagic acid	y = 4.81*x* + 0.02	1.0000	1-250	0.07	0.22	1.17	1.5	2.05	1.80
Chlorogenic acid	y = 5.00*x* + 0.20	0.9999	1-250	0.14	0.43	0.74	1.2	1.75	1.09
Asiatic acid	y = 16.20*x* + 0.25	0.9999	0.5-100	0.08	0.25	0.95	1.24	2.06	1.17
Ferulic acid	y = 40.09*x* + 0.05	1.0000	0.5-100	0.02	0.07	0.65	1.1	1.85	1.10
Arjunic acid	y = 0.35*x* + 0.07	0.9996	10-500	0.66	1.99	1.19	1.69	2.54	1.68
Gallic acid	y = 41.46*x* + 0.02	1.0000	0.1-100	0.01	0.03	0.25	0.95	1.9	1.80
Paclitaxel	y = 309x+1.97	0.9993	5-500	0.02	0.06	0.63	1.2	1.80	0.96
Boswellic acid	y = 595x+2.91	1.0000	10-500	0.01	0.03	0.26	0.93	1.7	1.7

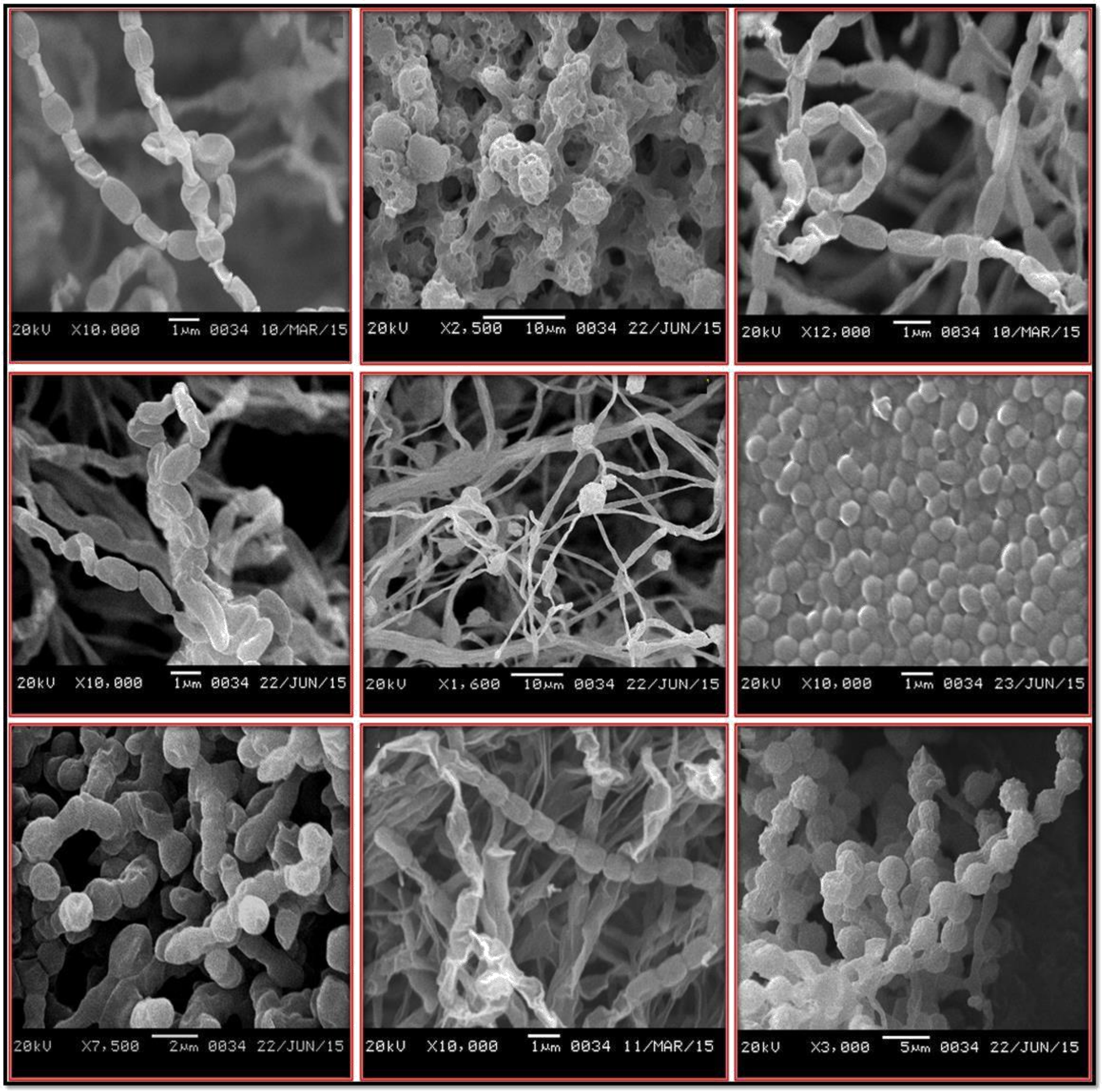

Supplementary Fig.S1 Scanning electron microscope showing spore chain morphology of endophytic actinobacterial isolates after 2 weeks at 28 ^{o}C
